# Cathepsin B is a New Drug Target for Traumatic Brain Injury Therapeutics: Evidence for E64d as a Promising Lead Drug Candidate

**DOI:** 10.3389/fneur.2015.00178

**Published:** 2015-09-02

**Authors:** Gregory Hook, J. Steven Jacobsen, Kenneth Grabstein, Mark Kindy, Vivian Hook

**Affiliations:** ^1^American Life Science Pharmaceuticals, Inc., San Diego, CA, USA; ^2^AstraZeneca Neuroscience iMed, Cambridge, MA, USA; ^3^Department of Chemical Engineering, University of Washington, Seattle, WA, USA; ^4^Department of Neurosciences, Medical University of South Carolina, Charleston, SC, USA; ^5^Ralph H. Johnson Veterans Administration Medical Center, Charleston, SC, USA; ^6^Skaggs School of Pharmacy and Pharmaceutical Sciences, University of California San Diego, La Jolla, CA, USA; ^7^Department of Neurosciences, Department of Pharmacology, University of California San Diego, La Jolla, CA, USA

**Keywords:** traumatic brain injury, cathepsin B, protease, E64d, drug, therapeutics

## Abstract

There is currently no therapeutic drug treatment for traumatic brain injury (TBI) despite decades of experimental clinical trials. This may be because the mechanistic pathways for improving TBI outcomes have yet to be identified and exploited. As such, there remains a need to seek out new molecular targets and their drug candidates to find new treatments for TBI. This review presents supporting evidence for cathepsin B, a cysteine protease, as a potentially important drug target for TBI. Cathepsin B expression is greatly up-regulated in TBI animal models, as well as in trauma patients. Importantly, knockout of the cathepsin B gene in TBI mice results in substantial improvements of TBI-caused deficits in behavior, pathology, and biomarkers, as well as improvements in related injury models. During the process of TBI-induced injury, cathepsin B likely escapes the lysosome, its normal subcellular location, into the cytoplasm or extracellular matrix (ECM) where the unleashed proteolytic power causes destruction via necrotic, apoptotic, autophagic, and activated glia-induced cell death, together with ECM breakdown and inflammation. Significantly, chemical inhibitors of cathepsin B are effective for improving deficits in TBI and related injuries including ischemia, cerebral bleeding, cerebral aneurysm, edema, pain, infection, rheumatoid arthritis, epilepsy, Huntington’s disease, multiple sclerosis, and Alzheimer’s disease. The inhibitor E64d is unique among cathepsin B inhibitors in being the only compound to have demonstrated oral efficacy in a TBI model and prior safe use in man and as such it is an excellent tool compound for preclinical testing and clinical compound development. These data support the conclusion that drug development of cathepsin B inhibitors for TBI treatment should be accelerated.

## Introduction

Traumatic brain injury (TBI) occurs when an external force, such as that due to a vehicular accident, a football collision, or a bullet, causes brain dysfunction and pathology. Unfortunately, TBI is all too common with over 10 million people worldwide afflicted each year (1) and at least 1.7 million cases annually in the United States where it is a leading cause of death among the young and the elderly (2, 3). Falls are the primary cause of TBI among the very young and old, whereas auto accidents and sports injuries are the main cause in 15- to 24-year-olds and most of those occur in males (2, 4). Military casualties add to these numbers with an overwhelming number of those due to blast injury and most of those are males (5).

TBI encompasses a continuum of injuries and pathologies and is symptomatically classified into mild, moderate, or severe based on the level of patient consciousness (6, 7). Much is known about the complex consequences of TBI (8, 9) including the necrotic and apoptotic neuronal cell death that occurs (10) and the TBI-related pathologies, which include, for example, inflammation (11), breakdown of vascular walls (12), ischemia (13), subarachnoid aneurysms and hemorrhages (14), brain edema (15), inflammatory pain (16), increased intracranial pressure (ICP) (17), infections (18), and neuroexcitatory toxicity (19). Moreover, sufficiently severe or repetitive TBI results in increased risks for many age-related neurological diseases, including amyotrophic lateral sclerosis (ALS) (20), Parkinson’s disease (PD) (21), Alzheimer’s disease (AD) (22), epilepsy (23), and possibly multiple sclerosis (MS) (24). As such, TBI can also be viewed as a model of neurodegenerative diseases generally (25).

But despite this knowledge, there is currently no pharmaceutically effective treatment for TBI even though many experimental clinical trials have been conducted over decades to find such (26–28). This may be because the drug targets, which affect outcomes, have yet to be identified and exploited and thus there is a continuing need for new TBI therapeutic targets.

Proteases and small molecule protease inhibitors are a proven target and means for successful pharmaceutical intervention as such inhibitors are approved for drug use to treat hypertension (29), HIV infection (30), and multiple myeloma cancer (31). The cysteine protease, cathepsin B, is a potential drug target for several diseases (32), including, for example, various cancers (33–36), pancreatitis (37), liver fibrosis (38), rheumatoid arthritis (RA) (39, 40) viral Ebola (41), bacterial *Streptococcus pneumoniae* meningitis (42), and parasitic *Trypanosoma cruzi* infections (43). While no cathepsin B inhibitor has yet been approved for drug use, one has completed Phase 1 trials for fatty liver disease (44, 45) and another is in late preclinical stage for treating Chagas disease (46).

However, cathepsin B’s potential as a TBI drug target has received relatively little attention with the last review to discuss this protease in a TBI context published a decade ago (47). Since then, cathepsin B gene knockout data have clearly demonstrated that cathepsin B is a significant cause of the behavioral dysfunction and pathology that occurs in animal models as a result of TBI (48). This review summarizes the evidence showing that genetically eliminating or pharmaceutically reducing cathepsin B activity produces improved outcomes in animal models of TBI, other types of trauma, and the many TBI-related pathologies mentioned above. The review also focuses on the small molecule, E64d and its derivatives, as a tool compound for developing a TBI lead therapeutic because of its demonstrated efficacy by many groups by many routes of administration in many TBI and TBI-related animal models and its prior safe use in man. The paper includes a basic introduction to the enzymology, biology, distribution, regulation, and function of cathepsin B and how increased expression and redistribution of cathepsin B from lysosomes to the cell cytosol and extracellular matrix (ECM) likely causes the cathepsin B-induced pathology. The overall conclusion drawn is that cathepsin B is an important target for treatment of TBI and that E64d and its derivatives have many of the preclinical properties needed for a successful TBI therapeutic agent (49–51) and should be developed for such.

## Cathepsin B Properties: Enzymology, Genetics, Transcription, and Translation

### Cathepsin B enzymology

Cathepsin B is among the most studied proteases as there are numerous reports written over the last 76 years. Its proteolytic activity was first identified in beef tissue (52). Originally called cathepsin II (53), it was renamed cathepsin B 63 years ago (54) and was purified 5 years after that (55). The first amino acid sequences were determined 32 years ago (56), and the first human, rat, and mouse genes were cloned 3 years later (57). The first X-ray crystal structure was resolved almost 25 years ago (58). While the first report on cathepsin B gene-deficient mice was made 17 years ago (59), it was not until last year that such animals were evaluated for improving TBI deficits (48).

#### Endopeptidase and Exopeptidase Activity

Most proteases have either endopeptidase or exopeptidase activity. Cathepsin B is unusual in having endopeptidase activities (60) as well as peptidyl-dipeptidase (61) and carboxypeptidase (62) exopeptidase activities. Its multi-enzymatic capability is due to a unique structural element of the protein called the “occluding loop” (58). At low pH, as occurs in the lysosome, the loop closes on the enzymatic active site and does not allow polypeptide binding and thereby reduces endopeptidase activity but permits peptidyl-dipeptidase and carboxypeptidase activities. At higher pH, the loop opens from the active site and allows more endopeptidase activity (63). The multiple activities of cathepsin B make it particularly well suited among proteases for digesting unwanted proteins, but also make it potentially more destructive in TBI brain damage and pathology.

#### Selectivity of Peptide Cleavage Sites

**Figure 1:**
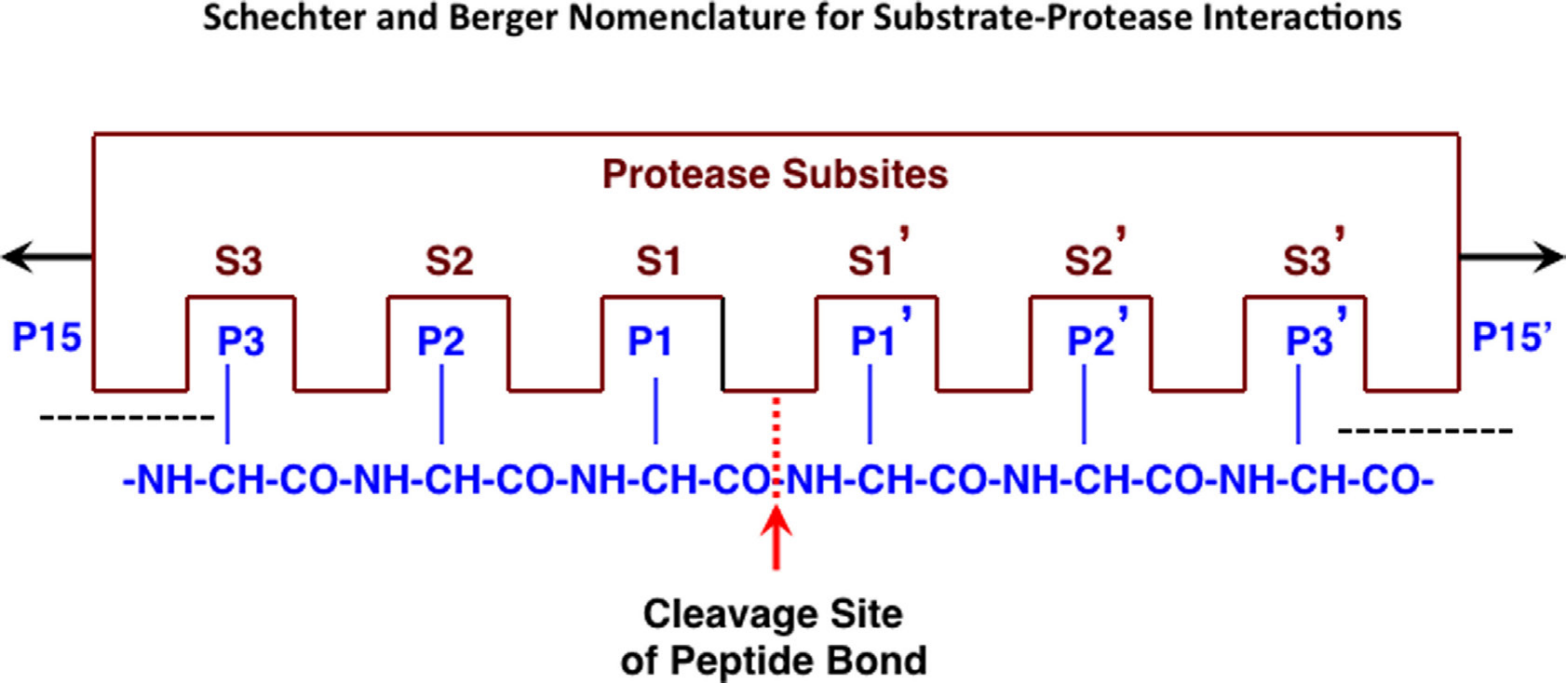
**Protease interactions with polypeptide substrates for proteolysis**. This figure illustrates protease and polypeptide substrate interactions utilizing the Schechter and Berger nomenclature (68). The active site of a protease is composed of several subsites. The scheme shows an active site of six subsites, termed S1–S3 and S1′–S3′. Subsites are located on both sides of the catalytic site and are numbered from there in either direction. The positions of the amino acid residues of the peptide substrate represent their locations from the cleaved peptide bonds and have the same numbering as the subsites they occupy (P1–P3 and P1′–P3′). Cleavage occurs between P1 and P1′ residues. Figure revised from Ref. (69).

Endopeptidases recognize specific amino acid sequences flanking the scissile peptide bond of the cleavage site (64) (see Figure 1). A small peptide substrate containing the recognition sequence can mimic the recognition site in proteins and can be used to monitor protease activity. Using such substrates, cathepsin B has been shown to prefer positively charged arginine (Arg) or lysine (Lys) at the P1 position, which is an unusual recognition signal among proteases, and a bulky hydrophobic amino acid residue at P2 (65, 66). Cathepsin B will also accept a basic residue, Arg, at the substrate P2 position (67). Thus, the substrate Z-Arg-Arg-AMC (where Z = benzyloxycarbonyl) with AMC (amido-4-methylcoumarin hydrochloride) is commonly used to assay cathepsin B activity *in vitro* as its cleavage liberates fluorescent AMC that monitors protease activity. While the recognition motif is not absolute, significant changes in the types of amino acids adjacent to the cleavage site can drastically affect endopeptidase binding and cleavage.

#### Classification

Proteases are classified by catalytic type, which are composed of aspartic, cysteine, metallo, serine, and threonine, and a small group of uncertain catalytic type. Cathepsin B has a reactive cysteine residue within its catalytic domain and belongs to the cysteine catalytic class. Proteases are further classified according to their clan, which is based on the homology of the three-dimensional structure, the arrangement of catalytic residues in the active site, and the amino acid sequence around the catalytic site. Cathepsin B is a member of the CA clan and shares a common structure with the dyad reactive cysteine and histidine residues at specific positions within the catalytic site (70). They are further classified into families in which they are grouped together with proteases having a statistically significant relationship in the amino acid sequence to a representative member type. The cathepsin B sequence is highly homologous to the plant cysteine protease papain (56), which is the reference protease of the papain-like family. Thus, cathepsin B is a cysteine protease belonging to the CA clan and the papain-like family, C1A. The other papain-like cysteine proteases in man are cathepsins F, H, C, K, L, O, S, V, W, and X. Enzymes as a whole are also systematically classified according to the Enzyme Commission (EC) based on a numerical code related to the type of activity catalyzed by the enzyme but not on homology and the enzyme code for cathepsin B is EC3.4.22.1.

Common protease names, which are often based on their discovery and not on catalytic type or homology, can cause confusion. For example, the name “cathepsin,” which is derived from the Greek katehepsin (to digest), was given to the protease activity in an acidic environment. Those proteases were subsequently found to be of different catalytic types and include the serine proteases cathepsins A and G, the aspartic proteases cathepsins D and E, and the lysosomal papain-like cysteine proteases (70, 71).

### Cathepsin B genetics

In man, cathepsin B is encoded as a single gene on chromosome 8 at position p22-23.1 (72), spans 27 kilobases, and contains 13 exons (73, 74). Interestingly, the gene location of cathepsin B on chromosome 8p is near a potential hub for development of neuropsychiatric disorders and an area of high divergence between chimpanzee and man (75). Human cathepsin B gene polymorphisms are associated with the disease tropical calcific pancreatitis (76). In mouse, cathepsin B is also encoded as a single gene on chromosome 14 at position 33.24, spans 20 kilobases, and contains 10 exons and 9 introns (77, 78). The human and mouse nucleic acid sequences encoding the proteolytically active form have 82% homology (57).

### Cathepsin B transcription

Human cathepsin B messenger ribonucleic acid (mRNA) consists of multiple messages that differ in their 5′ and 3′ untranslated regions (UTRs) and arise by alternative splicing. Most tissues express 2.3 and 4.0 kilobase (kb) transcripts at a ratio of 2:1; but the ratio of mRNAs with variant 5′ UTRs differs widely (74). Normal human brain contains both cathepsin B mRNA transcripts but most is the 2.3 kb form (79).

Small, non-coding microRNA (miRNA) inhibit the expression and function of endogenously encoded proteins (80). The miRNA expression patterns vary among organs (81), are altered by central nervous system (CNS) injuries, and the changes vary among spinal cord contusion (SCC), stroke, and TBI injuries (82). TBI causes at least 35 and 50 miRNAs to be up- and down-regulated, respectively, and their predicted targets involve signal transduction, transcription, proliferation, and differentiation (83). miR-218 and miR-128 down-regulate cathepsin B expression when they are up-regulated in medulloblastoma cell lines (84) and AD monocytes and lymphocytes (85) and are expressed in brain neurons (81, 86).

### Cathepsin B translation

Cathepsin B post-translational processing is well understood and has been the subject of many reviews (60, 70, 87). Human cathepsin B is initially transcribed as a preproenzyme of 339 amino acids. The 17 amino acid pre-signal sequence is co-translationally removed and the resultant proenzyme is translocated to the Golgi-apparatus where aspargine becomes glycosylated by a high-mannose-type sugar. The mannose-6-phosphate residues target the proenzyme to the lysosomes. The preproenzye or proenzyme forms are not enzymatically active. Activation of the proenzyme occurs with cleavage of the 62-residue pro region, resulting in mature cathepsin B. The mature form (30 kDa) is enzymatically active and can be further processed by removal of the C-terminal 6 amino acids and the excision of a dipeptide resulting a heavy chain (25 kDa) and a light chain (5 kDa). The heavy and light chains form a 2-chain form (30 kDa) linked together by a cysteine–cysteine bond. The heavy and 2-chain forms are enzymatically active. In some cells, such as neuroendocrine cells, cathepsin B is also trafficked to the regulated secretory pathway (RSP) but how that occurs is not known. The cathepsin B prepro, pro, and mature amino acid sequences of mouse and man are 65%, 74%, and 82% homologous, respectively (57). Normal human brain contains inactive precursor cathepsin B and proteolytically active forms (79).

Proteases are stored in the cell as inactive zymogens that require hydrolysis for activation, and allow cells to rapidly deploy active protease as needed (88). In the case of cathepsin B, the inactive proenzyme is hydrolyzed by a variety of proteases, including the aspartic protease cathepsin D, other cysteine proteases, and cathepsin B self-cleavage to liberate the mature active form (89–91). Maximum cathepsin B zymogen activation occurs at an acidic pH and very little occurs at a neutral pH (91), but cathepsin B zymogen can endure a neutral pH and be subsequently activated in an acidic environment (60). Mature cathepsin B is degraded by cysteine protease nicking (87, 92).

## Traumatic Brain Injury and TBI-Related Injuries Activate Cathepsin B

### PreClinical data: Cathepsin B in TBI and TBI-related animal models

#### TBI Increases Cathepsin B Expression in Animal Models

As shown in Table 1, cathepsin B mRNA, protein, and activity are increased following trauma in TBI animal models. Trauma significantly up-regulates brain cathepsin B mRNA expression about 2.5-fold relative to controls at 3 days after TBI in fluid percussion injury rat and controlled cortical impact (CCI) mouse models (93). Trauma elevated cathepsin B protein levels and activity, using a weight drop mouse model, in the cortex at the injury site and in the hippocampus distal to the injury site beginning as soon as 6 h; the elevated brain cathepsin B continued for at least 1 week with a 75% maximum increase occurring at about two days post-trauma (94, 95). In a severe TBI rat model, brain trauma elevated brain cathepsin B protein levels within 1 h and those levels remain elevated for up to 32 days after injury with a 300% maximum increase at 8 days post injury (96). Our data in a severe CCI mouse model show a 100% and 380% increase in brain cathepsin B activity and protein levels at 2 and 24 h post-trauma, respectively (48). Trauma elevates cathepsin B protein levels 1 day to 3 days post injury in a moderate closed-skull TBI rat model and this increase varied with injury location (97). The prolonged elevation of cathepsin B after trauma suggests that it may be possible to therapeutically intervene clinically, as treatment often does not begin until several hours after injury. The fact that different groups using different moderate and severe TBI animal models find increased cathepsin B following injury shows that higher cathepsin B expression is a consistent response in rodent TBI models.

**Table 1:** **Cathepsin B is activated in TBI and TBI-related animal models**.

Animal model (species)	Cathepsin B			Reference
	mRNA	Protein	Activity	
Trauma TBI (mouse, rat)	↑	↑	↑	(48, 93, 94, 96–98)
Trauma SCC (rat)	↑	↑	↑	(99, 100)
Trauma surgery post-op ileus (mouse)	nd	nd	↑ in ECM	(101)
Subarachnoid hemorrhage (rat)	nd	↑	nd	(102, 103)
Brain aneurysm (rat)	↑	nd	↑	(104)
Chronic hypertension brain edema (rat)	nd	↑	nd	(105)
Acute ischemic edema (rat)	nd	↑	nd	(106)
Brain ischemia (monkey, rat)	nd	↑	↑	(106–110)
Neuroexcitatory epilepsy (rat)	nd	↑	nd	(111)
Neuroexcitatory Huntington’s disease (rat)	nd	↑	nd	(112)
Infection brain meningitis (mouse)	nd	nd	↑	(42)
Infection sepsis (rat)	nd	nd	↑	(113, 114)
Inflammation/pain (mouse)	nd	↑	nd	(115)
Inflammation/aging (mouse)	↑	↑	nd	(116)
Neurodegenerative ALS transgenic (mouse)	↑	nd	nd	(117, 118)
Neurodegenerative AD Transgenic 5XFAD (mouse)	↑	nd	nd	(119)
Neurodegenerative AD Transgenic APPSwe/PS1 (mouse)	nd	↑	nd	(120)

*nd, not done; ECM, extracellular matrix; ALS, amyotrophic lateral sclerosis; 5XFAD, express APP containing three familial AD mutations (FAD) and human PS1 containing two FAD; APPSwe/PS1, express human APP containing one FAD and human PS1 containing 2 FAD*.

#### TBI-Related Animal Models

Table 1 shows that cathepsin B expression is increased in several TBI-related injuries in animal models. SCC is related to TBI in that both are trauma to the CNS and in a SCC rat model, cathepsin B mRNA, 37 kDa proprotein, 30 kDa mature protein, 25 kDa mature protein, and activity increased 20-, 3.5-, 4.5-, 10-, and 7-fold, respectively, at the injury site 7 days after trauma relative to sham animals (99, 100). Surgery is a form of trauma and in a surgical post-operative ileus mouse model, cathepsin B activity increased twofold in the ECM on the day after trauma relative to control (101) and was accompanied by a loss of the ECM microvascular basal lamina and collagen-type IV as is characteristically observed in TBI (12). Thus, other trauma models also show a similar increase in cathepsin B expression as seen in TBI models.

TBI causes many vascular pathologies including ischemia, aneurysms, subarachnoid hemorrhages, and edema (13–15). Increased cathepsin B expression has long been known to result from ischemia with brain activity increasing about two- and fivefold relative to controls at 2 and 5 days after injury in ischemic rat and monkey models, respectively (107–109). Similarly, in an aneurysm model, cathepsin B mRNA increased about fivefold and activity increased about twofold 3 months after aneurysm induction relative to controls (104). In a subarachnoid hemorrhage model, cathepsin B protein increased about 3.5-fold at 2 and 3 days after hemorrhage induction relative to controls (102, 103). Cathepsin B protein levels are increased in chronic hypertension brain edema (105) and acute ischemic edema models (106). Thus, cathepsin B expression is also increased in important vascular pathologies caused by TBI.

Brain infections are a common TBI complication (18) and cathepsin B levels are greatly increased in meningitis and sepsis animal models (42, 113, 114). Moreover, aseptic inflammation and pain are major factors in TBI (16), and cathepsin B is elevated in aseptic inflammatory and pain animal models (115, 116).

Finally, as discussed above, TBI is a risk factor for several neurodegenerative diseases. Cathepsin B mRNA is elevated 3.5-fold relative to controls in spinal cord samples from an ALS mouse model (117). Moreover, an ALS meta-analysis of the literature identified cathepsin B as repeatedly up-regulated in ALS patients and mouse models (121). TBI causes excitotoxicity (19) and cathepsin B protein levels are elevated in excitotoxicity animal models of recurring epilepsy and Huntington’s chorea (112, 122). Furthermore, cathepsin B mRNA is increased in the transgenic AD 5Xfamilial AD (FAD) mouse model, which expresses human amyloid precursor protein (APP) containing three familial AD mutations and human presenilin 1 (PSN1) containing two familial mutations (119). Cathepsin B protein is also increased by 50% in the cortex and hippocampus of the APPSwe/PS1 model, which expresses human APP containing the Swedish (Swe) FAD mutation and PS1 with FAD mutations, relative to controls (120). Thus, cathepsin B is elevated in several neurodegenerative animal models associated with TBI.

### Clinical data: Cathepsin B regulation in TBI-related injuries

#### Short-Term Changes in Cathepsin B Observed in Polytrauma and Aneurysm

We are not aware of any clinical studies on acute cathepsin B regulation due to TBI, but non-brain polytrauma patients show increases in plasma cathepsin B activity during the first day after trauma, which subsequently falls to moderately elevated levels by the third day, and remains roughly at that level for up to 2 weeks. Importantly, the increase in plasma cathepsin B activity correlates with the severity of injury. Patients with a sixfold increase in plasma cathepsin B activity 1 day post-trauma subsequently developed fatal or reversible multiple organ failure, whereas those who had only a threefold increase, at the same day, did not display organ failure (123, 124).

Moreover, in human cerebral aneurysm tissue, cathepsin B is highly expressed in the endothelial cell layer and the media in the aneurysmal walls in contrast to control artery tissue where it is barely expressed at all (104). Thus, cathepsin B expression appears to be significantly increased as a result of TBI type injuries in man.

#### Long-Term Changes in Cathepsin B in Chronic Inflammatory Conditions

Patients having the chronic inflammatory neurological diseases Guillain–Barre syndrome, chronic demyelinating polyneuropathy, or MS have higher cerebrospinal fluid (CSF) cathepsin B activity levels than controls (125, 126). Cathepsin B mRNA levels in ALS postmortem spinal cord tissue has been shown to be about 2- and 3.3-fold higher than that of non-neurological age-matched controls (117, 127) and cathepsin B protein expression is increased and has an abnormal distribution, especially in the anterior horn, relative to controls (128). AD brain autopsy samples show a high cathepsin B protein expression, especially near pathological amyloid plaque brain deposits, relative to age-matched control samples (129). Moreover, AD patients have a significant 50% higher plasma and serum cathepsin B protein level than age-matched control samples (120, 130) and higher serum cathepsin B levels in AD patients strongly correlates with reduced cognitive ability (120). Interestingly, cathepsin B protein levels in peripheral blood lymphocytes and monocytes from AD patients are about 50% lower than those from controls (85) and that, taken together with the plasma and serum data, suggest that cathepsin B may redistribute from peripheral blood cells to the serum/plasma compartment in AD patients. CSF cathepsin B studies in AD patients have shown a significant increase (131) by proteomic analysis and no significant difference but a trend toward higher levels by ELISA and Western blot analysis relative to controls (130, 132).

In the peripheral inflammatory conditions of RA and osteoarthritis (OA) synovial cells and chondrocytes have increased cathepsin B mRNA levels and cathepsin B protein secretion relative to controls (133, 134). Furthermore, in patients with inflammatory bowel disease, cathepsin B is up-regulated in areas of tissue damage and mucosal ulceration (135).

## Cathepsin B Gene Knockout Improves Deficits in TBI and TBI-Related Animal Models

### Cathepsin B knockout mice are healthy

The health of cathepsin B gene knockout mice is maintained and generally indistinguishable from normal littermates in behavior, histology, and fertility (136), as shown by data summarized in Table 2. The only reported difference is a decrease in thyroglobulin (Tg) solubilization (137) but this does not appear to cause a significant phenotypic or behavioral effect. The normal health of mice lacking cathepsin B implicates that pharmacologic inhibition of cathepsin B will likely be generally safe.

**Table 2:** **Animals lacking the cathepsin B gene are healthy**.

Behavior	Morphology/histology	Fertility	Biomarkers	Reference
nd	nd	nd	No effect on MHC antigen processing	(59)
No apparent behavioral deficits	Indistinguishable from wild-type mice	Normal	Normal	(136)
nd	nd	nd	Reduced thyroglobulin solubilization and degradation	(137)

### TBI mice with cathepsin B knockout: Improved deficits and pathology

**Figure 2:**
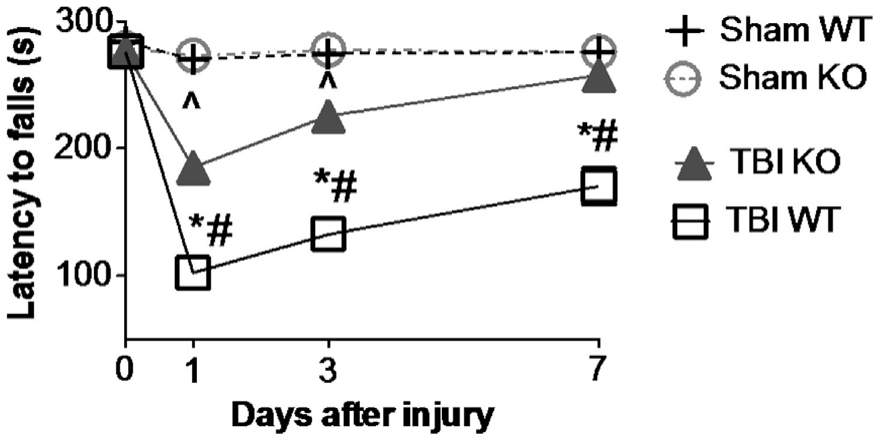
**Cathepsin B gene deletion improves neuromotor deficits caused by TBI**. Mice with knockout of the cathepsin B gene were assessed for TBI-caused neuromotor deficits. Four groups of mice were assessed: sham wild-type (Sham WT), sham cathepsin B gene knockout (Sham KO), TBI WT, and TBI cathepsin B gene knockout (TBI KO). TBI was modeled by controlled cortical impact (CCI) and mice were subjected to rotarod behavioral neuromotor evaluations before and 7 days after TBI trauma. Longer latency times indicate better neuromotor function. Sham WT and Sham KO animals were not surgically treated the same as TBI animals and were not traumatized. Significantly, knockout of the cathepsin B gene resulted in improved neuromotor defictis and a shorter recovery period compared to TBI WT mice (mean ± SEM, Bonferroni’s multiple comparison test *P* < 0.05, *N* = 10 animals/group, *TBI WT vs. Sham WT, ^TBI KO vs. Sham KO, and ^#^TBI WT vs. TBI KO) (48). Data from cited publication adapted for graphic display.

**Figure 3:**
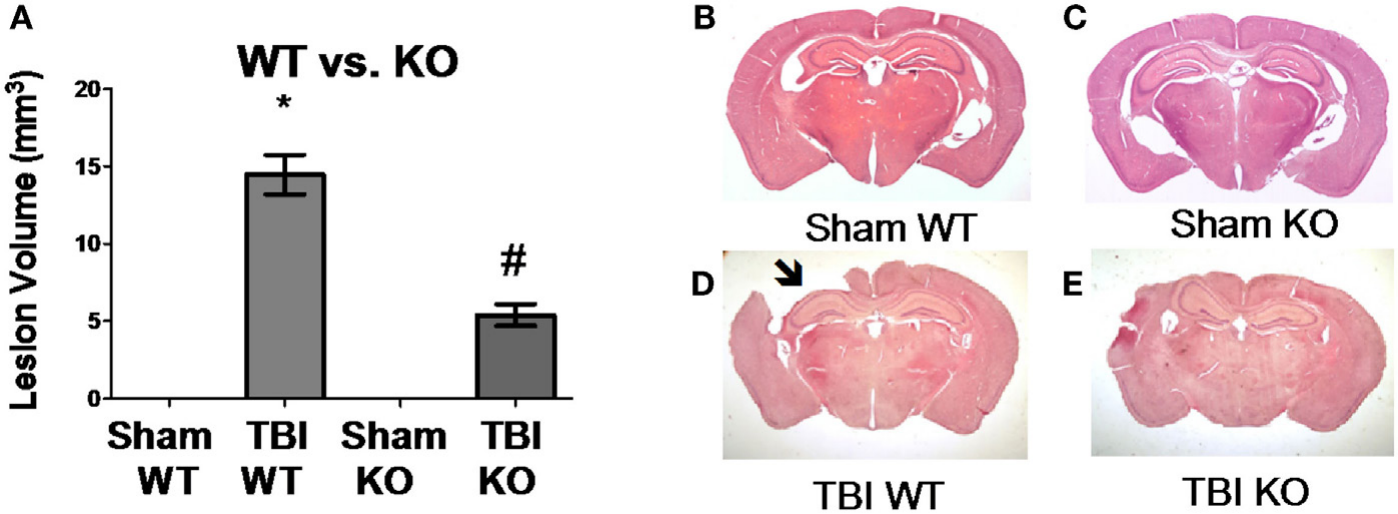
**Cathepsin B gene deletion reduces brain tissue lesions caused by TBI**. At 7 days post-TBI (mice receiving CCI TBI), mouse brains were evaluated to determine the brain volume loss (Lesion Volume) at the impact site. **(A)** Quantitative image analysis of brain sections showed that cathepsin B gene deletion minimized brain tissue loss. The Sham WT and Sham KO animals had no loss whereas the TBI WT mice had significant loss, but the TBI KO mice had roughly one-third the loss suffered by the TBI WT mice. Representative micrographs from the brains of Sham WT, Sham KO, TBI WT, and TBI KO animals are shown in **(B–E)**, respectively. (mean ± SEM, Bonferroni’s multiple comparison test *P* < 0.05, *N* = 10 animals/group, *TBI WT vs. Sham WT, Sham KO, and TBI KO, and ^#^TBI KO vs. Sham WT and Sham KO) (48). Data from cited publication adapted for graphic display.

**Figure 4:**
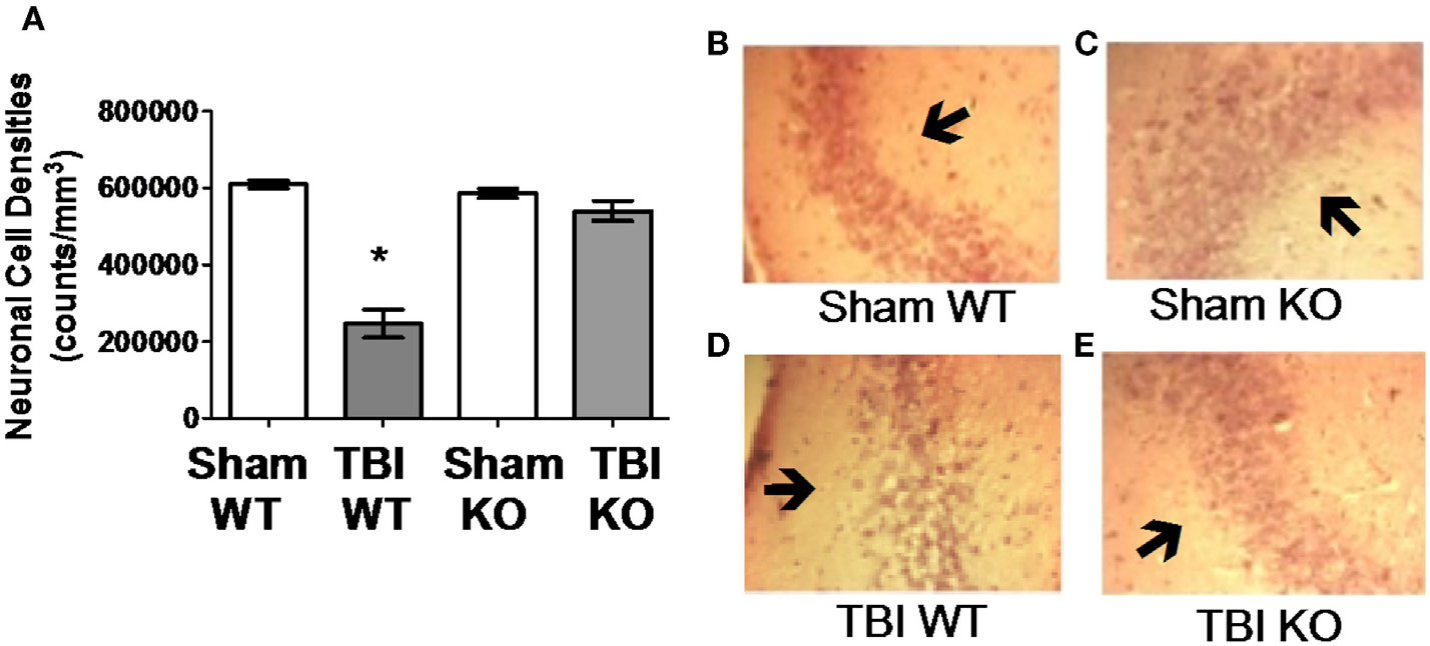
**Cathepsin B gene deletion reduces neuronal loss after TBI**. Quantitative image analyses of brain sections evaluated for Lesion Volume were also analyzed for neuronal cell density in the CA3 region of the hippocampus, which is distal to the impact site. **(A)** TBI WT, but not TBI KO, mice had lower neuronal density than Sham WT and Sham KO animals. Thus, cathepsin B knockout resulted in reduced neuronal loss. Representative micrographs from the brains of Sham WT, Sham KO, TBI WT, and TBI KO animals are shown in **(B–E)**, respectively (mean ± SEM, Bonferroni’s multiple comparison test *P* < 0.05, *N* = 10 animals/group, * TBI WT vs. Sham WT, Sham KO and TBI KO) (48). Data from cited publication adapted for graphic display.

Table 3 summarizes the data on the effect of deleting the cathepsin B gene in TBI models and shows that the deletion improves behavior, pathology, and neuronal cell survival (48). Figure 2 shows that deleting the cathepsin B gene reduces the severity and duration of the neuromotor dysfunction that occurs during the week following trauma with the deficient mice suffering about half the dysfunction of wild-type (wt) mice 1 day after trauma and regaining normal function, whereas the wt mice remain significantly impaired 1 week after trauma. Figures 3 and 4 show that 1 week after trauma, cathepsin B gene-deficient animals have about one-third the brain lesion volume of wt animals and no hippocampal neuronal cell loss, whereas wt mice suffered a 60% loss relative to the sham animals. As discussed below, apoptosis contributes to TBI-induced cell death and the pro-apoptotic cell death biomarker, Bax, is elevated 1 day post-trauma in wt mice, but not in cathepsin B gene knockout mice relative to sham controls. The clear conclusion is that cathepsin B is a significant contributor to the behavioral dysfunction and neuronal cell loss that follows TBI.

**Table 3:** **Cathepsin B gene deletion improves deficits of TBI and TBI-related animal models**.

Model	Cathepsin B gene deletion effect			Reference
	Behavior	Pathology	Biomarkers	
Trauma TBI	↓ Neuromotor dysfunction	Brain ↓ Lesion vol; Neuron death	↓ Brain bax	(48)
Trauma surgery post-op ileus	nd	↓ ECM destruction	↓ ECM collagen IV	(101)
Neuroexcitatory epilepsy	No effect on seizures	↓ Brain neuron death	nd	(138)
Neurodegeneration AD transgenic human APPwt	nd	nd	Brain ↓ Aβ (1-40/42); CTFβ ↑ sAPPα	(139)
Neurodegeneration disease AD transgenic human APPLon	↓ Memory deficits	↓ Brain Aβ plaque	Brain ↓ Aβ(1-40/42); CTFβ ↑ sAPPα	(140)
		↓ Brain pGlu-Aβ plaque	↓ Brain pGlu-Aβ(3-40/42)	(141)
Neurodegeneration AD transgenic human APPSwe	nd	↑ Brain plaque	No significant change brain Aβ (1-X/42)	(142)
Neurodegeneration fibrial Aβ, chromogranin microglia challenge	nd	nd	↓ microglia IL-1β, caspase 1	(143)
Inflammation/pain	↓ Chronic inflammatory pain	↓ Activated microglia	Brain ↓ mIL-1β; mIL-18; Cox2	(115)
Aging inflammation	nd	nd	↓ Brain IL-1β	(116)
LPS-induced inflammation	nd	nd	Macrophages ↓ TNFα secretion	(144)
TNFα challenged hepatocytes	nd	nd	Hepatocytes ↓ caspase 2 ↓ mito cyt c	(145)
Neuro-degeneration MS EAE cathepsin B and S double knockout	nd	Improved clinical score ↓ spinal cord leukocyte infiltration ↑ age of onset	↓ Immune cell markers (MHC-II, CD69 CD4+ cells)	(146)

*nd, not done; ECM, extracellular matrix; mIL-1β, mature interleukin-1β; mIL-18, mature interleukin-18β; mito cyt c, mitochondrial cytochrome c; ALT, alanine aminotransferase; HSC, hepatic stellar cells; APPwt, wild-type amyloid precursor protein; APPLon, amyloid precursor protein containing the London mutation, CTFβ, C-terminal β-secretase fragment; pyrogluAβ, pyroglutamate Aβ; EAE, experimental autoimmune encephalomyelitis. Blue and tan colors indicate significant effects on behavior and pathology, respectively, by cathepsin B gene deletion*.

### TBI-related models with cathepsin B knockout show improved outcomes

Deleting the cathepsin B gene produces significant beneficial outcomes in TBI-related pathological animal models and includes surgery, epilepsy, AD, inflammation, pain, and cytokine cell death models. Deletion of both cathepsin B and cathepsin S improves the outcomes in an MS model (see Table 3).

#### Surgery Model

In the traumatic post-operative ileus surgery model, deleting the cathepsin B gene results in significantly less ECM breakdown and collagen-type IV loss than occurs in wt animals in that model (101). Cathepsin B, therefore, is likely a key target to preventing ECM breakdown resulting from TBI.

#### Epilepsy Model

TBI increases the risk of epilepsy (23), which can result in neuroexcitotoxicity. Deleting the cathepsin B gene in the Unverricht–Lundborg progressive myoclonus epilepsy model prevents about 90% of the apoptotic neuronal cell death that usually occurs in these animals. (138). Thus, cathepsin B is also likely to be an important target for preventing the TBI neuroexcitotoxity-induced cell death.

#### Alzheimer’s Disease Model

TBI increases the risk of AD (22), which is thought to result from the abnormal accumulation of brain amyloid-β (Aβ). In transgenic AD mice expressing human APP containing the human wt β-secretase site, which is what most AD patients have, deleting the cathepsin B gene improves memory deficits and reduces the brain amyloid plaque, which is a neuropathological hallmark of AD, that develop in these animals (140, 141). That deletion in such transgenic animals also reduces brain Aβ(1-40/42) and the pernicious post-translationally modified pyroglutamate (pGlu) Aβ(3-40/42) forms (139–141), which are thought to be particularly neurotoxic among Aβ peptide species (147). These and other data (148–150) show that cathepsin B has wt β-secretase activity, which cleaves APP and can produce Aβ species.

On the other hand, cathepsin B gene deletion in transgenic models expressing APP containing the Swe FAD mutation does not significantly affect Aβ and increases amyloid plaque (142). The Swe mutation alters the amino acid sequence at the β-secretase site, which destroys the cathepsin B target sequence and thus cathespsin B does not cleave this form of APP (151). However, the Swe FAD mutation occurs in only one extended family, whereas most people express normal APPwt. Thus, cathepsin B gene deletion reduces Aβ in models mimicking the β-secretase activity occurring in most humans. Given that, the inhibition of cathepsin B may be able to prevent the increased brain Aβ, which also occurs after TBI (152, 153).

#### Inflammation and Inflammatory Pain Models

TBI induces microglia activation and inflammation, which can occur for a prolonged period after trauma and can cause neuronal cell death (154). Moreover, TBI causes inflammatory pain (16) and increases the proinflammatory cytokines interleukin-1β (IL-1β), and interleukin-18 (IL-18), which are related to pain (155, 156). These cytokines are controlled by caspase 1 in protein complexes known as inflammasomes, which are also activated by TBI (157). Administering Freund’s adjuvant to animals, in the peripheral tissues, induces inflammatory pain and inflammation, but doing so to cathepsin B gene-deficient mice results in significantly less pain (tactile allodynia), less IL-1β, IL-18, caspase 1 activation, and less inflammasome activation than occurs in wt animals (115, 143). Microglia phagocytosis of fibrillar Aβ or chromogranin A (CGA) causes microglia activation and cathepsin B expression leading to inflammasome formation and production of IL-1β, and activated caspase 1. These microglia responses do not occur in cathepsin B-deficient mice (116, 158). These data suggest that cathepsin B is also a critical target for reducing CNS inflammation and inflammatory pain caused by TBI.

#### TNFα Models

Tumor necrosis factor alpha (TNFα) is a cytokine that induces a cytoplasmic molecular cascade including cathepsin B release from lysosomes and apoptotic cell death (159) and TBI increases brain TNFα (160). Cathepsin B gene-deficient mice are resistant to TNFα-induced liver damage and hepatocyte apoptotic cell death and have reduced TNFα-induced caspase activation and mitochondrial cytochrome c (cyt c) release, which are key apoptotic proteins (145, 161). More recently, macrophages from cathepsin B-deficient mice treated with lipopolysaccharide (LPS), which induces an intense TNFα response, were shown to secrete 50% less TNFα than wt macrophages (144). Thus, cathepsin B is an important target for preventing the TNFα-induced cell death that occurs as a result of TBI.

#### Cathepsin B and Cathepsin S Knockouts have Improved Outcomes in Multiple Sclerosis Model

TBI has been associated with an increased risk of subsequent MS development in the Chinese population (24). In an experimental autoimmune encephalomyelitis (EAE) mouse model of MS, deletion of cathepsin B or cathepsin S gene alone had no effect but deletion of both cathepsin B and S genes improved clinical scores and significantly delayed age of disease onset relative to sufficient animals (146).

### Summary: Cathepsin B knockout has little effect in development but has major neuroprotective effects on TBI and TBI-related injuries

The cathepsin B gene knockout mice show little or no adverse impact on normal functions, but have major beneficial effects in TBI, including reductions in neuromotor deficits, brain pathology, and neuronal cell death. Cathepsin B deficiency produces substantial improvement in important pathologies related to TBI, including reductions in ECM breakdown, neuroexcitatory-induced cell death, inflammation, inflammatory pain, TNFα-induced cell death, Aβ levels, and memory deficits.

It is likely that the absence of developmental defects associated with cathepsin B deficiency is due to redundant protease specificity of the closely related protease, cathepsin L, which can substitute for the normal function of cathepsin B. Evidence in support of this hypothesis is that cathepsin B and L double knockouts are lethal and have profound neurological abnormalities (162, 163). Thus, some cysteine cathepsin proteolytic activity is required for fetal development. This has implications, which are discussed below (7.5), for the therapeutic development of compounds.

The large volume of data from the cathepsin B knockout mice indicates that this protease is central to many different pathological processes. Many benefits occur from this deficiency, leading to the compelling conclusion that cathepsin B inhibition is a promising therapeutic approach for TBI.

## Mechanisms of Cathepsin B Regulation in Normal Compared to TBI Injury Conditions

### Neurobiology of normal cathepsin B regulation

#### Cathepsin B Distribution

##### Tissue distribution of cathepsin B

**Figure 5:**
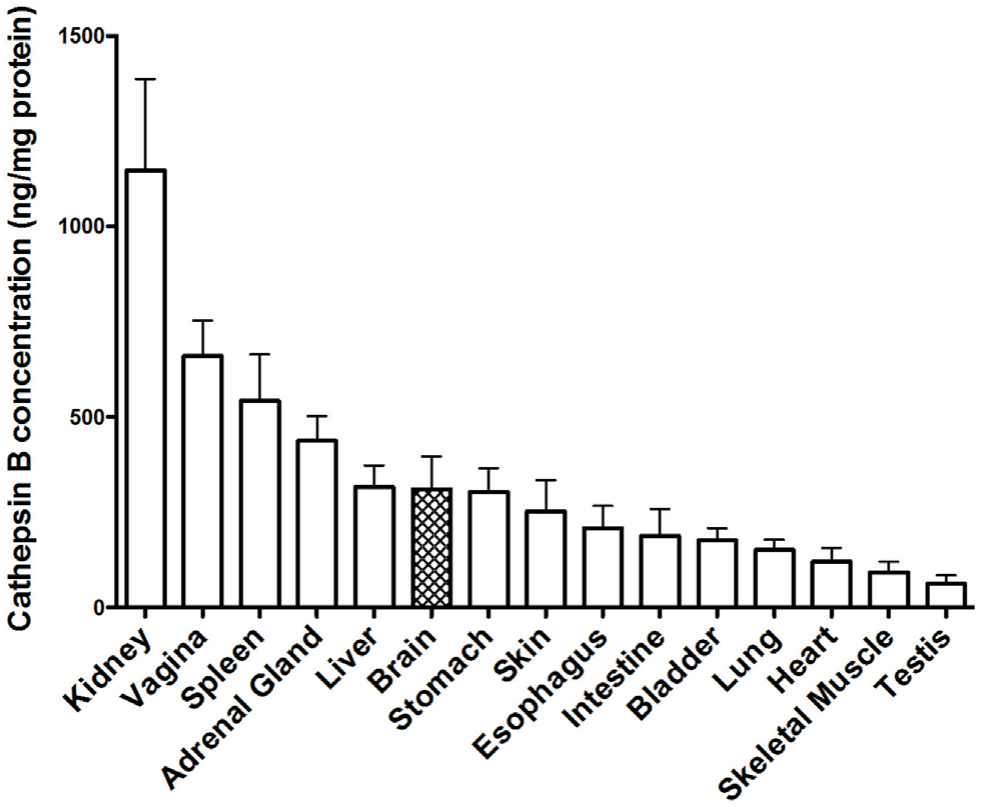
**Cathepsin B protein concentration vary among tissues**. The levels of mature cathepsin B concentrations are shown from rat tissues (mean ± SD displayed, *N* = 4 analyses, means significantly different, ANOVA, *p* < 0.0001) (165). Data from the cited publication and adapted to graphic display and analyzed for variance.

Early studies demonstrated that cathepsin B is present in human tissues throughout the body (164), but that the concentration varied as exemplified in Figure 5, which shows the cathepsin B concentrations in selected rat tissues (165). Moreover, these studies also established that cathepsin B concentrations varied among cell types with rat peripheral macrophages having cathepsin B concentrations that are 33, 50, and 400 times higher than lymphocytes, neutrophils, and erythrocytes, respectively, and that immunologically activating macrophages causes a further sixfold increase in cathepsin B levels (165). The cathepsin B variation among the different tissues and cells suggests that cathepsin B is differentially expressed among the tissues and has specialized functions in addition to lysosomal protein degradation (165, 166).

An on-line human protein tissue atlas (167) also shows that cathepsin B is widely expressed in the body with cathepsin B mRNA found in all and protein detected in 80% of the tissues (http://www.proteinatlas.org/). Moreover, the amount of cathepsin B expression varies among different tissues and cell types.

##### Brain cathepsin B

Early immuno-microscopic analysis showed that human brain contains cathepsin B and it is concentrated in neuronal cell types, especially in the hippocampus (164, 168–171). By contrast, only a few glia cells in normal brain were seen to contain significant amounts of cathepsin B (168). A similar intense neuronal cell staining was also seen in rodent brain, especially in the pyramidal cells of the cortex, large neurocytes of the septal region, many hippocampal neurons, and magnocellular nerve cells of the hypothalamus (97, 168, 172, 173).

**Figure 6:**
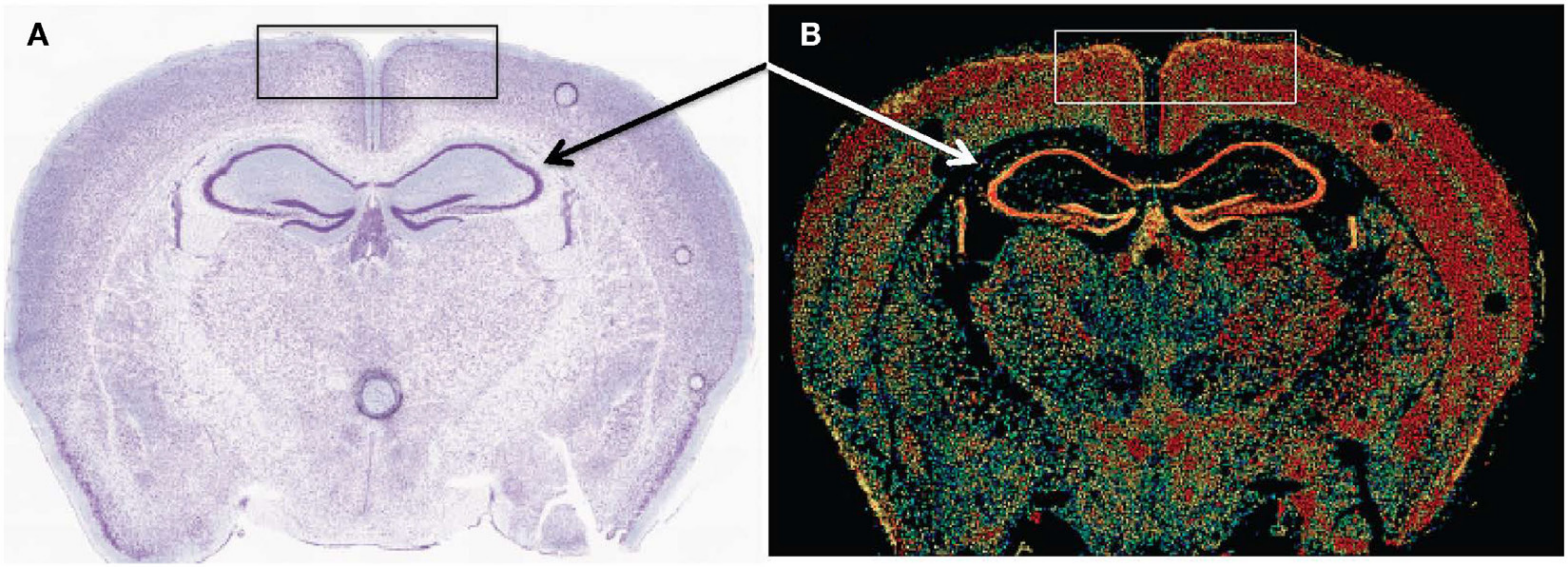
**Cathepsin B expression occurs in selected regions of the brain**. **(A,B)** are micrographs of the same coronal mouse brain section and show tissue structure and cathepsin B mRNA expression, respectively. In **(A)**, the section is nissl stained, which highlights neurons as dark blue. In **(B)**, *in situ* hybridization of sections with antisense mRNA to cathepsin B illustrates the brain regions of cathepsin B mRNA expression. Hotter colors, such as yellow and red, signify high expression, cooler colors, such as green and blue, indicate low expression, and black indicates undetectable expression. A comparison of the two micrographs shows that cathepsin B is intensely expressed in the hippocampal neuronal cell layer (arrows) and in the cortex (box). Figures taken from the Allen Brain Institute web site http://www.brain-map.org/.

Early cathepsin B mRNA analysis of rat brain also showed cathepsin B expression concentrated in neurons (174) and more recent data confirm this distribution. Figure 6 shows cathepsin B mRNA expression and histological images in a normal mouse brain section. Cathepsin B is selectively and intensely expressed in the hippocampal neuronal cell layer and in the cortex. The images were obtained from the Allen Brain Institute web site. A comparison of the papain-like cysteine protease mRNA expression levels in that section shows that cathepsin B is expressed the most followed by cathepsin L with all others expressed at very low levels or not at all.

##### CSF and plasma cathepsin B

Table 4 shows the human CSF and plasma cathepsin B concentrations (130). The plasma concentration is about 14 times greater than that in the CSF. Human and rat CSF cathepsin B concentrations increase with age (175, 176).

**Table 4:** **Human CSF and plasma cathepsin B concentrations**.

Sample (number of individuals)	Measured concentration^c^ ng/mL	Estimated molar concentration^d^ **μ**mol/L	Reference
CSF (118)^a^	8.4	3.36 × 10^−4^	(175)
CSF (28)^b^	9.6 ± 3.4	3.84 × 10^−4^	(130)
Plasma (28)^b^	134.3 ± 71.3	53.7 × 10^−4^	(130)

*^a^Surgery patients, 21–82 years old, mean age 65 years, predominately male*.

*^b^Healthy volunteers, mean age 63.9 ± 6.2 years old, predominately female*.

*^c^Value reported by reference, mean ± SD*.

*^d^Estimated value to facilitate comparison with Table 5 data assuming a cathepsin B molecular weight of 25,000*.

#### Subcellular Organelle Compartments and Functions

##### Lysosomes

A major destination for cathepsin B is the lysosome, the name of which is from the Greek lysis: loosen and soma: body. Christian de Duve discovered that these subcellular structures were recycling centers in which unwanted proteins are dismantled to their amino acid building blocks for recycling into new proteins for which he received the 1974 Nobel Prize in Physiology or Medicine (177). Lysosomes are membrane-bound compartments present within neurons and cells in the cytosol but outside the nucleus. Lysosomes sequester cytosolic proteins at a rate of no more than 4% per hour but rapidly digest those with a half-life of less than 10 min, which means that the proteolytic capability is at least 20 times that of the highest substrate uptake (178, 179). This rapid hydrolysis is due to the high lysosomal protease concentration, which can be more than 1 mM (180), present at a pH significantly more acidic than the cytosol of the cell (discussed in the section below on pH and cystatin). Lysosomal cathepsins L, B, and S account for roughly 50% of the total bulk protein degradation (181) and the lysosomal cathepsin B concentration is about 330 ng/mg protein (87). Thus, cathepsin B is a major and key component of lysosomes, which are powerful proteolytic machines containing high concentrations of proteases capable of rapid digestion of large amounts of proteins.

In addition to digestive lysosomes, cathepsin B is also trafficked to secretory lysosomes, which release their contents to the extracellular environment in response to a triggering event. These lysosomes are primarily found in immunological cell types and brain astrocytes (182, 183). Containment in and release from secretory lysosomes to the extracellular space allows the cell to safely export the proteolytic capability of cathepsin B and other lysosomal proteases as needed. For example, glia cells secrete cathepsin B in response to neurotoxin exposure via secretory lysosomes (184).

But lysosomal cathepsin B can also cleave proteins at specific recognition sites to produce protein fragments having important biological activities. For example, cathepsin B cleaves in the lysosome non-phosphorylated myristoylated alanine-rich C kinase substrate (MARCKS), which is the primary substrate for protein kinase C (PKC), to prevent phosphorylation and thereby control cell morphology and motility (185–187). In the thyroid gland, cathepsin B traffics via secretory lysosomes to the extracellular lumen of the thyroid follicles (188) where it cleaves Tg and solubilizes it (137).

##### Autophagosomes

Autophagosomes are structures in which unnecessary or dysfunctional cellular components are degraded. Lysosomes fuse with autophagosomes to provide the proteases needed for that degradation and cathepsin B is a key protease in autophagy catabolism (189).

##### Regulated secretory vesicles

Lysosomes are not the only subcellular destination for cathepsin B (190). It is also found in regulated secretory vesicles of cells, which secrete large amounts of enzymes, hormones, or neurotransmitters to the extracellular space. Neurons and endocrine cells are such cells and are unique in having a RSP (regulated secretory pathway), in which material to be exported is densely stored in regulated secretory vesicles that discharge their contents out of the cell in response to a triggering signal (191). In pancreatic acinar cells, more cathepsin B is present in the RSP than lysosomes, and in the RSP cathepsin B cleaves trypsinogen to activate trypsin (192, 193). Cathepsin B is in the RSP of β-cells of mouse pancreatic islets where it co-localizes with insulin (194). Cathepsin B is found in dense secretory granules of kidney juxtaglomerular cells (195). In bovine adrenal chromaffin cells, cathepsin B is present in the RSP where it proteolytically cleaves wt APP to produce Aβ (148). As in the lysosome, cathepsin B is physically separated from the cytosol by membranes of secretory vesicles within the RSP.

##### Summary: Cathepsin B is Normally Packaged into Discrete Subcellular Organelles

From a TBI point of view, the important point is that cathepsin B is normally mostly contained within subcellular organelles and this compartmentalization allows the cell to safely utilize the proteolytic powers of cathepsin B without damaging the cell structure. As discussed below, that segregation is compromised after brain trauma leading to cathepsin B-induced injury and pathology.

#### pH and Cystatin Control of Cathepsin B and Cysteine Protease Activity

##### pH regulation

Cathepsin B zymogen is activated by cathepsins in the lysosome/endosome compartments where they have optimum efficiency in the acidic environment of about pH 5.0 and the papain-like cysteine proteases require a reducing environment (67). The lysosomal membrane contains molecular pumps, which create that environment inside the lysosome and maintain it against the neutral pH 7.4 of the cytosol.

Cathepsin B’s enzymatic activity is reduced at neutral pH (196). For example, cathepsin B cleaves trypsinogen at pH 4.0 to 5.2, but not at a higher pH (192). Nonetheless, cathepsin B retains significant activity at the neutral pH of the cell cytosol after translocation from the lysosomes (197). Cathepsin B has a complex pH dependency among its endopeptidase and exopeptidase activities. *In vitro*, cathepsin B exopeptidase activity predominates below pH of 5.5 and endopeptidase activity prevails above pH 5.5 (198). Glycosaminogycans bind to cathepsin B to stabilize its structure and preserve its endopeptidase activity in the neutral pH of the cytosol (199). In comparison, cathepsin B is much more stable than cathepsin L at neutral pH (200). The key point is that cathepsin B retains significant enzymatic activity at pH 7.4 and thus can inflict major damage when set loose in the cytosol or the extracellular environment.

##### Cystatin regulation

The endogenous cystatin proteins are probably the most important means of controlling cytosolic and extracellular cysteine protease activity (200); cystatins are emergency inhibitors that neutralize cysteine proteases, which escape from the lysosomes (70, 201) and are of high importance in TBI. Type 1 cystatins are primarily intracellular proteins and type 2 cystatins are found in extracellular fluids. Cystatin C, a type 2 cystatin, is the controlling inhibitor for cathepsin B in human extracellular fluids, including CSF, blood plasma, synovial fluid, milk, saliva, seminal fluid, amniotic fluid, and tears (202). The inhibitor concentrations vary among the fluids but cystatin C has the highest CSF concentration of any of the endogenous inhibitors and is the most potent cathepsin B inhibitor of the endogenous inhibitors in those fluids. The cathepsin B activity half-life (t_1/2_) when exposed to cystatin C at the concentrations in the extracellular fluids is less than a second (202).

Table 5 shows the human CSF and plasma cystatin C concentrations (203). The CSF cystatin C concentration is about 5.5 times higher than that in plasma. Comparing the molar concentrations with those for cathepsin B in Table 4 shows that the CSF and plasma cystatin C concentrations are 16,000 and 170-fold greater than that of CSF and plasma cathepsin B concentrations, respectively. The high cystatin C to cathepsin B ratios show that inhibiting cathepsin B activity in these compartments is critical, especially in the brain. TBI increases cystatin C and an increase in cystatin C soon after trauma is associated with reduced neuronal cell damage (97).

**Table 5:** **Human CSF and plasma cystatin C concentrations**.

Sample (number of individuals)	Measured concentration^b^ **μ**mol/L	Reference
CSF (28)^a^	5.6 ± 1.6	(130)
Plasma (28)^a^	0.9 ± 0.2	(130)

*^a^Healthy volunteers, mean age 63.9 ± 6.2 years old, predominately female*.

*^b^Value reported by reference, mean ± SD*.

### Lysosomal leakage of cathepsin B

Lysosomal release of cathepsin B to the cytoplasm can overwhelm the normal controls and cause significant damage. The potential of the lysosome as a “suicide bag” capable of killing the cell if its protease contents escape to cytosol and other compartments has long been recognized (204).

#### TBI Causes Lysosomal Leakage of Cathepsin B

Trauma causes lysosomal cathepsin B leakage to the cytosol in animal models. In normal brain, immunostaining shows that all neurons have a punctate cathepsin B distribution, which reflects its lysosomal location, but after TBI injury many neurons display diffuse staining throughout the neuron cell body showing that cathepsin B has escaped the lysosome containment (97, 205).

Controlled cell shear of primary neuronal cell cultures mimics TBI damage and causes neuronal cell death (206). Cytosolic and lysosome fractions from sheared cells have elevated and reduced cathepsin B, respectively, which is consistent with lysosomal cathepsin B leakage. Sheared neurons are more likely to die than unsheared neurons and treatment with the membrane sealant Poloxamer 188 prevents leakage and reduces cell death in sheared cells (206). Moreover, Poloxamer 188 treatment in TBI CCI and ischemic mouse models reduces neuronal cell death (207, 208).

#### TBI-Related Injuries also Cause Lysosomal Leakage of Cathepsin B

Ischemia causes the rearrangement of cathepsin B from the lysosomes to the cytoplasm in non-human primate brain neurons (108). Niemann–Pick disease Type C is a genetic lysosomal storage disease, and in mouse models of this disease, cathepsin B leaks out of lysosomes in neurons of the cerebellum (209). In epilepsy animal models, seizures result in translocation of lysosomal cathepsin B to the cell body and nucleus (210). ALS autopsy samples show that cathepsin B is diffusely distributed within degenerative neurons (128). In nerve cells of brain autopsy tissue from AD and Parkinson’s dementia of Guam and senile dementia, but not age-matched controls, cathepsin B is found in neurites and dendrites and in the pathological neurofibrillary tangles and plaque structures (129, 170, 171). And in AD and Parkinson’s cell culture models, Aβ(1-42) and α-synuclein cause lysosomal leakage of cathepsin B into the cytoplasm (211–213).

#### Mechanisms of Lysosomal Leakage of Cathepsin B

Molecular mechanisms of lysosomal leakage have been summarized in an excellent recent review (214). Many of the mechanisms were discovered in abnormal cancer cells and those are not discussed because it is not clear how relevant they are to normal neurons.

Unique to trauma is that mechanical force directly breaks membranes and is likely the primary cause of lysosomal rupture at the site of injury. These forces can rapidly damage large amounts of tissue and cause the release of significant amounts of proteases, which autodigest the brain tissue.

Ischemic monkey and rat animal model data generated the “calpain/cathepsin” neuronal cell death hypothesis, which is based on lysosomal cathepsin B leakage (215, 216). Injury induces calcium ion entry into neurons causing μ-calpain activation, which indirectly permeabilizes lysosomal membranes. Heat shock protein 70.1 (HSP 70.1) normally stabilizes lysosomal membranes by binding to them via endolysosomal phospholipid bis(monoacylglycero)phosphate (BMP) and enhances membrane stabilizing acid sphingomylinase (ASM) activity. Ischemia decreases HSP 70.1 and BMP and increases oxidized HSP 70.1, which is cleaved by activated μ-calpain, and those changes decrease ASM and together cause lysosomal membrane ­permeability, the release of lysosomal cysteine proteases, and cell death (217).

Cathepsin B also contributes to lysosomal leakage as illustrated by the reduced lysosomal leakage that occurs in cathepsin B-deficient hepatocytes vs. wt cells responding to TNFα toxicity (218). The fact that trauma causes increased cathepsin B and TNFα suggests that cathepsin B may contribute to lysosomal leakage in TBI.

Free radical formation can also contribute to lysosomal membrane breakdown. TBI pathology causes a pronounced increase in free radicals and oxidative brain damage (219, 220). The free radicals enter lysosomes, react with iron to form hydroxyl radicals, which react with lysosomal membrane components thereby destabilize the lysosomal membrane and cause it to leak (221). Treating subarachnoid hemorrhage models with iron chelators deferoxamine or α-lipoic acid protected lysosomal membranes and prevented cathepsin B leakage, which correlated with improved outcomes, such as reductions in brain edema, blood–brain-barrier impairment, and neuronal cell death, and also improved behavioral deficits (102, 103).

### TBI-induced lysosomal leakage causes necrotic and apoptotic cell death, inflammation, and axonal damage

#### Necrotic Cell Death

The extent to which the lysosomal membrane is disrupted by an injury is a key driver in determining the resulting pathology. A frank rupture is generally thought to result in necrotic cell death whereas a lesser amount of leakage causes apoptotic cell death (222, 223). The molecular machinery causing necrotic and apoptotic death is complex and diverse and has been the subject of several reviews (214, 222, 224).

The molecular mechanisms by which cathepsin B participates in necrotic cell death are yet to be fully understood presumably because of the difficultly in studying the rapidly occurring multiplicity of reactions that lead to necrotic death. But one mechanism by which lysosomal rupture causes necrotic cell death is through activation of the inflammasome receptor NOD-like receptor (NLR) family pyrin domain-containing 3 (NLRP3) by cathepsin release into the cytoplasm (225). As discussed below, NLRP3 activation by cytosolic cathepsin B is also central to the inflammatory response caused by lysosome leakage.

#### Apoptotic Cell Death

Apoptotic cell death cascade occurs following trauma and cathepsin B is a key component of that cascade (94, 98). TNFα is elevated after trauma and it is an extracellular cell death signal that sets into motion intracellular molecular events that ultimately lead to cell death. The pathway includes TNFα binding to tumor necrosis factor receptor 1 (TNFR1) causing cytosolic activation of caspase 8, Bid cleavage to t-Bid, and polymerization of BAX, which forms mitochondrial membrane pores causing mitochondrial leakage to the cytoplasm of cytochrome C (cyt c), which activates the apoptotic initiator caspase 9 causing activation of the executioner caspase 3 (226). Direct evidence that cathepsin B contributes to this pathway in TBI comes from data showing that deleting the cathepsin B gene in a TBI mouse model blocks BAX activation following trauma (48). E64d, which is an inhibitor of cathepsin B and other papain-like cysteine proteases and calpains 1 and 2 discussed below (see section on ‘E64, E64d, and E64c cysteine protease inhibitors’), administered to TBI mouse models reduced proapoptotic proteins t-Bid, BAX, and cytosolic cyt c, and activated caspase 3 and antiapoptotic protein Bcl-2 (94). The effects of E64d on reducing apoptotic cell death are likely primarily due to cathepsin B inhibition because E64d given to cathepsin B knockout mice produced no additional reduction on BAX levels after trauma than that obtained in cathepsin B gene deletion mice given the vehicle solution (48). Thus, cathepsin B is an important component to TBI-induced TNFα cell death.

Studies in hepatocytes from cathepsin B knockout animals that show the TNFα-induced lysosomal leakage of cathepsin B to the cytosol cleaves Bid and activates caspase 2, which facilitate mitochondrial release of cyt c to the cytosol and that triggers caspase apoptotic cell death (145, 161). Treatment of normal human blood lymphocytes with antithymocyte antibodies also results in lysosomal cathepsin B leakage to the cytosol and apoptotic cell death by an undefined mechanism that does not involve the intrinsic mitochondrial cyt c pathway (227). Unraveling the exact means by which cathepsin B functions in the TBI-induced death pathway will be an exciting area of future research.

#### Inflammation

Cathepsin B is secreted by inflamed microglia and induces apoptosis. Trauma to the CNS causes diverse inflammatory cells expressing high levels of cathepsin B, which include microglia and macrophages, to gather at a site of injury (100). Inflammation induced by CGA in primary microglia cultures causes those cells to secrete cathepsin B, which induces apoptotic cell death in primary granule neuronal cultures possibly by executioner caspase 3 activation (228). Thus, cathepsin B in the extracellular space can induce neuronal apoptotic cell death.

TBI can cause massive cellular and cytokine inflammatory responses that focus on cathepsin B as the center of TBI-caused damages. As discussed above, cathepsin B is clearly involved in the production of IL-1β by activating caspase 1, which in turn cleaves pro-IL-1β to produce active IL-1β (116) as has been shown in microglia inflamed by fibrillar Aβ or CGA or in alveolar macrophages inflamed by silica. Interestingly, lysosomal permeabilization and NLRP3 activation are required for fibrillar Aβ- (158) and silica- (229) induced inflammations, but not in the CGA-induced inflammation (115). Alternatively, cathepsin B can also activate IL-1β by cleaving pro-caspase 11 to activate proinflammatory caspase 11 (230), which in turn can cleave pro-caspase 1 (231). As such, cathepsin B can control IL-1β activation by multiple mechanisms. Moreover, cathepsin B also increases TNFα secretion in response to LPS-induced inflammation by functioning in the trafficking of vesicles containing TNFα (144). Thus, cathepsin B both contributes to TNFα levels and transduction of the TNFα apoptotic cell death signal. Cathepsin B also activates IL-18 levels in response to CGA-induced inflammation (115).

Peroxisome proliferator-activated receptor (PPAR) receptors are key regulators of neuroinflammation after CNS injury, including TBI (232). PPARα agonist fenofibrate is effective in TBI models (233, 234). PPARδ agonists suppress cathepsin B levels in human endothelial cells in a PPARδ-dependent manner (235). Thus, PPARδ agonists target cathepsin B and may be beneficial in TBI because they reduce cathepsin B.

#### Axonal Damage

Pathological axonal damage is a predictor of outcome in CNS diseases (236) and cathepsin B may be involved in causing that damage. As mentioned above, cathepsin B cleaves MARCKS, which is the PKC substrate that controls cell morphology and motility by regulating actin dynamics near the cell surface. N-Methyl-d-aspartate (NMDA) treatment of primary hippocampal neuronal cell cultures mimics excitotoxicity, increases cathepsin B, reduces MARCKS at synapses, and causes dendritic spine collapse. Treating with CA-074Me, which is a cathepsin B inhibitor discussed below, prevents spine collapse in the NMDA-treated neurons. (186, 237). Thus, activated cathepsin B cleavage of MARCKS may be a mechanism that contributes to axonal swelling.

### TBI-induced autophagy

TBI increased autophagy activation within 1 h and up to at least 3 days after trauma in the brain relative to non-traumatized controls as measured by lipidated microtubule-associated protein light chain 3 (LC3II) (94, 238–241) with higher LC3II levels suggesting autophagy activation (242). Moreover, beclin 1, which interacts with phosphatidylinositol-3-kinase (Class III PI3K) to induce autophagy (243), was also elevated 1 h and up to at least 3 days post-TBI in the cortex and hippocampus relative to controls (239–241). Furthermore, microscopic examination shows more cellular autophagy structures after trauma than before (238, 239). Autophagy proteolysis was also increased post-TBI as measured by P62 with less P62 generally reflecting more autophagy proteolysis (244), as P62 was reduced within 1 h and up to at least 2 days post-trauma in the cortex and hippocampus relative to controls (240, 241).

#### Inhibiting Autophagy Improves TBI Outcomes

While it has been suggested that autophagy might provide neuroprotection after TBI (239), administration of the autophagy inhibitor 3-methyladenine (3-MA) to TBI animal models reduced the TBI-increased LC3II and Beclin 1 levels and restored the reduced P62 levels that resulted from trauma, while improving memory and neuromotor defects and reducing brain lesion volume, neuronal cell death, cathepsin B activity, and caspase 3 activity (241). 3-MA reduces autophagy by inhibiting Class III PI3K and does not inhibit cathepsin B activity (245). Moreover, treating animals with the gamma-glutamylcysteinyl ether ester, which is a prodrug of glutathione and an antioxidant, also reduced autophagy and improved TBI outcomes (238). These data suggest that reducing autophagy improves TBI outcomes. Cathepsin B inhibitors inhibit lysosomal cysteine proteases, which reduces autophagy function (246) and thereby may also improve TBI outcomes.

## Small Molecule Inhibitors of Cathepsin B Improve Behavioral and Pathological Deficits in TBI and TBI-Related Animal Models

### Inhibitors of cathepsin B improve deficits of TBI animal models

Table 6 summarizes the data showing the behavioral, pathological, and biomarker effects of administering small molecule inhibitors of cathepsin B to TBI and TBI-related injury animal models. These include models of ischemia, subarachnoid and cerebral hemorrhage, meningitis, pain, and neurodegenerative conditions including epilepsy, AD, MS, and Huntington’s disease. Table 7 summarizes the protease inhibition profiles of the small molecules cited in Table 6.

**Table 6:** **Small molecule inhibitors of cathepsin B are efficacious for improving behavioral and pathological deficits in TBI and TBI-related animal models**.

Disease model	Compound (method)	Compound effects (relative to control)			Reference
		Behavior	Pathology	Biomarkers	
Trauma TBI	E64d (oral, A)	↓ Motor deficits	Brain ↓ Lesion vol; neuronal death	Brain ↓ Cat B; Bax	(48)
	CA-074Me (icv, B)	↓ Memory deficits	Brain ↓ Lesion vol; neuronal death	Brain ↓ Cat B act; t-Bid; Bax; mito cyt c; caspase 3	(94)
	z-DEVD-fmk (icv, A)	↓ Motor and cognitive deficits	Brain ↓ Lesion vol; Neuronal death	Brain ↓ Calpain I act; LDH; α-spectrin degradation	(247)
	LHVS (icv, B)	↓ NNS ↑ Grip strength	Brain ↓ Edema; neuronal death	Brain ↓ TNFα; IL-1β	(248)
Trauma SCC	E64d (iv, A)	nd	Spinal cord ↓ Reactive gliosis	Spinal cord ↓ Calpain act; GFAP; caspase 3; DNA fragments	(249)
Neuroexcitatory epilepsy	E64d (ip, B)	nd	↓ Brain mossy fiber sprouting	Brain mRNA ↓ Cat B; PRG-1; PRG-3; PRG-5; ApoE; Clusterin; nSMase ↑ ANX7	(122)
	CA-074Me (ip, B)	↑ Neurological scores; learning ability	nd	Brain ↓ Cat B act; LC3II/LC3I; Beclin-1; Bcl-2; PRG-1	(111)
Neuroexcitatory huntington disease	Z-FA-FMK (is, B/A)	nd	↓ Brain lesion volume	nd	(112)
Pain	CA-074Me (it, B)	↓ Inflammatory pain	nd	Microglia: ↓ mIL-1β; mIL-18β	(115)
Infectious brain meningitis	CA-074Me (ip, B and A)	nd	Improved Clinical score; ↓ CSF WBC; ICP	↓ Brain IL-1β	(42)
Ischemia	E64c (ip, B)	nd	nd	↓ Brain MAP2 degradation	(250)
	CA-074 (iv, A)	nd	↓ Brain neuronal death	↓ Brain cat B	(108)
	CA-074; E64c (iv, A)	nd	↓ Brain neuronal death	↓ Brain cathepsin B activity	(109)
	CP-1 (iv, A)		↓ Brain infarct volume	nd	(251)
	CA-074, E64c (iv, A)	nd	↓ Brain neuronal death	nd	(252)
	E64d (ip, B)	nd	Brain ↓ Infarct vol; neuronal death; Edema; vascular damage	Brain: ↓ Cat B: Calpain I; Caspase 3; Vascular: ↓ Cat B; Calpain I; Caspase 3	(106)
	CP-1 (iv, A)	↓ Neurological defects	↓ Infarct vol.	Brain: ↓ Cat B act; Heat shock prot; Serum albumin; CRMP2	(110)
Cerebral aneurysm	NC-2300 (oral, A)	nd	Brain ↓ Aneurysms; ECM degradation	Aneurysm: ↓ Cat B act; Cat K act; Cat S act ↓ Collagenase; ↑ Elastin	(104)
Cerebral bleeding	CP-1 (iv, A)	↓ Motor sensor deficits	Brain ↓ Tissue loss; neuronal death ↑ Neuronal proliferation	Brain ↑ Synaptophysin; TUJI; Brd; VWF	(253)
Inflammation rheumatoid arthritis	E64d (ip B/A)	↓ Clinical symptoms	Joint ↓ pathology	Joint: ↓ IL-1β; IL-6	(40)
Inflammatory pain and edema	K11777 (ip B and A)	↓ Inflammatory pain	↓ Edema; Necrosis; pathology	↓ Cat B; Cat L; Cat S; Amylase act Spinal cord neurons: ↓ c-Fos	(254)
	CA-074Me (iv, B)	nd	↓ Pathology; edema	↓ Cat B act; Trypsin act; TAP; MPO act; Amylase act	(37)
Neuro-degenerative AD transgenic APPSwe	E64 (ip)	↓ Memory deficits	↑ Long-term potentiation	Brain Aβ(1-40/42) no change	(255)
Neuro-degenerative AD transgenic APPLon	E64d (oral) E64d (oral)	↓ Memory deficits	↓ Brain Aβ plaque ↓ Brain pGlu Aβ plaque	Brain ↓ Aβ(1-40/42); CTFβ ↑ sAPPα ↓ Brain pGlu Aβ(3-40/42)	(256) (141)
Neurodegenerative MS EAE	LHVS (ip)	nd	Improved clinical score ↓ Spinal cord leukocyte infiltration ↑ Age of onset	↓ Immune cell markers (MHC-II, CD69 CD4+ cells)	(146)

*B, A, treated before and after inducing pathology, respectively; icv, intracerebroventricular; ip, intraperitoneal; iv, intravenous; is, intrastriatal; it, intrathecal; NNS, neurological severity score; ICP, intracranial pressure; CSF WBC, cerebrospinal fluid white blood cells; PRG-2, 3, 5, plasticity related gene 2, 3, 5, respectively; ApoE, apolipoprotein E; sMase 2, spingomyelinase 2; ANX7, annexin 7; Clusterin, apolipoprotein J; MAP2, microtubule-associated protein; CRMP2, collagen response mediator protein 2; TUJ1, Class III Beta-tubulin; BrdU, bromodeoxyuridine; VWF, von Willebrand factor; MPO, myeloperoxidase; TAP, trypsinogin activation peptide; GDPH, glycerol-3-phosphate dehydrogenase; ALT, alanine aminotransferase; MIP2, macrophage inflammatory protein; KC, chemokine (CXC) ligand; αSMA, α smooth muscle actin; TGFβ, transforming growth factor β; COL1A1, collagen α1(I); TIMP, tissue inhibitor of metalloproteinases. Blue and tan colors indicate significant effects on behavior and pathology, respectively, by cathepsin B gene deletion*.

**Table 7:** **Protease inhibition profiles of small molecule inhibitors of cathepsin B, from studies of TBI and TBI-related animals**.

Name of inhibitor	Inhibition profile	Notes	Reference
**E64** (L-trans-epoxysuccinyl-leucylamido(4-guanidino)butane	Cathepsins B, L, H, K, S, F, O, V, W, X, calpains 1 and 2, and papain	Irreversible inhibitor	(70, 257–260)
**E64d** (EST, Loxistatin, ethyl (2S,3S)-3-[[(2S)-4-methyl-1-(3-methylbutylamino)-1-oxopentan-2-yl]carbamoyl]oxirane-2-carboxylate)	Cathepsins B, L, H, K, S, F, O, V, W, X, calpains 1 and 2, and papain	Clinical use Irreversible inhibitor	(261, 262)
**E64c** (Loxistatin acid, EP-475, (2S,3S)-3-[[(2S)-4-methyl-1-(3-methylbutylamino)-1-oxopentan-2-yl]carbamoyl]oxirane-2-carboxylic acid)	Cathepsins B, L, H, K, S, F, O, V, W, X, calpains 1 and 2, and papain	Clinical use Irreversible inhibitor	(261, 263)
**CA-074** (N-(L-3-trans-Propylcarbamoyloxirane-2-carbonyl)-L-isoleucyl-L-proline)	Cathepsin B (absolute specificity)	Irreversible inhibitor	(264–266)
**CA-074Me** (N-(L-3-*trans*-Propylcarbonyl-oxirane-2-carbonyl)-L-isoleucyl-L-proline methyl ester)	Cathepsins B and L	Irreversible inhibitor	(267–269)
**LHVS** (morpholinurea-leucine-homophenylalanine-vinylsulfone-phenyl)	Cathepsins S, B, and L	Irreversible inhibitor	(146)
**Z-FA-FMK** (methyl (3S)-5-fluoro-3-[[(2S)-2-[[(2S)-3-methyl-2(phenylmethoxycarbonylamino)butanoyl]amino]propanoyl]amino]-4-oxopentanoate)	Cathepsins B, L, and S; caspases 2, 3, 6, and 7; cruzain; and papain	Irreversible inhibitor	(112)
**Z-DEVD-FMK** (methyl (4S)-5-[[(2S)-1-[[(3S)-5-fluoro-1-methoxy-1,4-dioxopentan-3-yl]amino]-3-methyl-1-oxobutan-2-yl]amino]-4-[[(2S)-4-methoxy-4-oxo-2-(phenylmethoxycarbonylamino)butanoyl]amino]-5-oxopentanoate)	Cathepsins B and L, and caspases 3, 6, 7, 8, and 10	Irreversible inhibitor	(247, 270–272)
**CP-1** (O-benzyl, OBzl, carbobenoxy, Cbz-Phe-Ser(OBzl)-CHN2, 2[methyl-(4-phenylbenzoyl)amino]benzoic acid)	Cathepsins B and L	Reversible inhibitor	(110, 251, 253)
**K11777** (N-methyl-Piperazine-Phe-homoPhe-vinylsufone-phenyl)	Cathepsins B and L, and cruzain	Irreversible inhibitor	(43, 273)
**NC-2300** (VEL-0230, sodium (2S,3S)-3-(((S)-1-isobutoxy-4-methylpentan-2-yl)carbamoyl)oxirane-2-carboxylate)	Cathepsins K, B, and S	Irreversible inhibitor	(104)

*Short names of inhibitors are shown in bold. Parentheses indicate other names used for the inhibitor, including chemical names*.

The important conclusion from Table 6 is that different compounds, which inhibit cathepsin B, used by different groups in different TBI animal models resulted in improvements of neuromotor and cognitive defects, brain lesion volume, brain edema, neuronal cell death, and key brain biomarkers (48, 94, 247, 248). A particularly dramatic result is the significant reduction in lesion volume as the inhibitors protected against brain tissue loss much of which was likely due to necrotic cell death. Another major result was the prevention of neuronal cell death in the hippocampus as the inhibitors arrested nearly all the cell death that occurs in that region as a result of TBI. Behavioral dysfunction was also greatly improved by treatment with these compounds suggesting that it may be possible to also observe improved clinical outcomes with cathepsin B inhibitor treatment of TBI patients.

### Inhibitors of cathepsin B improve deficits in TBI-related conditions

Compounds, which inhibit cathepsin B, also produced substantial beneficial effects in TBI-related injuries in animal models. For example, neuromotor, neurological learning and cognitive defects were improved by cathepsin B inhibition in cerebral bleeding (253), ischemia (110), epilepsy (111), and AD neurodegeneration (256) animal models. Inflammatory pain is a major problem in TBI and was alleviated by cathepsin B inhibitors in inflammatory pain models (115, 254). Moreover, E64d treatment essentially eliminated the clinical symptoms of chronic inflammation due to RA (40) and greatly reduced the reactive inflammatory gliosis caused by the trauma of SCC (249).

Edema is often a major complication of TBI and compounds, which inhibit cathepsin B, reduced edema in ischemic (37, 106) and pancreatitis models (254). Excessive ICP and infections also often accompany TBI and treatment with a cathepsin B inhibitor reduced ICP, improved clinical scores, and lowered CSF white blood cell counts in a brain meningitis animal model (42). Brain aneurysms and vascular ECM degradation occur as a result of TBI, and cathepsin B inhibition stopped aneurysm progression and vascular ECM degradation in brain aneurysm (104) and ischemic (106) animal models. Brain tissue loss was significantly prevented by cathepsin B inhibitor treatment of cerebral bleeding (253), ischemia (106, 110, 251), and kainic acid-induced Huntington’s chorea animal models (112). Cathepsin B inhibitors have also been shown to prevent the vast majority of the neuronal cell death that occurs in ischemia (106, 108, 109, 252) and a great deal of the death resulting from cerebral bleeding animal models (253).

### Inhibition of cathepsin B modulates TBI biomarkers

The data in Table 6 also show that the inhibitors affected many TBI relevant biomarkers. TBI animal models treated with the cathepsin B inhibitors had reduced brain pro-apoptotic biomarkers including t-Bid, Bcl-2, Bax, cyt c, and caspase 3 (48, 94) and inflammatory proteins IL-1β and TNFα (248). The biomarker data show that cathepsin B inhibitors improve TBI outcomes in part by reducing apoptosis and inflammation.

Glia fibrillary acidic protein (GFAP), which is biomarker of CNS inflammation and a potential TBI diagnostic marker (274), is reduced by E64d treatment of SCC animals (249). Cathepsin B inhibitors also reduced cytokine IL-1β in pain, meningitis, and RA animal models (40, 42, 115), IL-18 in a pain animal model (115), and IL-6 in a RA model (40). As in the cathepsin B knockout data, cathepsin B inhibitors reduced brain Aβ(1-40/42) and pGlu Aβ(3-40/42) in transgenic AD mice expressing human APP containing the wt β-secretase site but not in those expressing the Swe mutant β-secretase site (141, 256).

These data support the conclusion that compounds, which inhibit cathepsin B, are effective in many TBI relevant animal models at improving behavior and pathology. As such, these data provide strong motivation to explore cathepsin B inhibitors as potential TBI therapeutic agents.

## E64d is a Promising Drug Inhibitor of Cathepsin B for Preclinical and TBI Therapeutic Drug Development

### E64d improves deficits of TBI and related injuries

#### TBI and Multiple Injury Conditions are Improved by E64d

Most of the preclinical and essentially all the clinical studies on small molecule inhibitors of cathepsin B investigated E64 and its derivatives E64d, E64c, CA-074Me, and CA-074. Table 6 shows that these compounds were efficacious in at least 17 different preclinical pathological models including two TBI models as well as one SCC, two epilepsy, two pain, one infectious, five ischemic, one arthritis, and three neurodegeneration (AD) models. Different dosing regimens, including administering the drug before or after the pathology onset, have been effective, as have different routes of administration, including oral, intraperitoneal (ip), intravenous (iv), intracerebroventricular (icv), and spinal intrathecal (it). Thus, there are robust data showing that E64 and its related compounds are efficacious in a wide range of TBI-related models using different treatment regimens and routes of administration. This is important because it is thought that potential TBI therapeutics must demonstrate efficacy in diverse animal models in order to have hope of success in treating the heterogeneity of TBI injuries in a clinical setting (275).

#### E64d TBI Efficacy is Primarily Due to Cathepsin B Inhibition, but Inhibition of Other Proteases Provides Added Benefits

Without additional data, it is difficult to conclude that the beneficial TBI effects of E64d are due to cathepsin B inhibition because the biologically active acid form of E64d, which is E64c, inhibits proteases in addition to cathepsin B, which are the papain-like cysteine proteases and calpains 1 and 2 (see section below on E64, E64d, and E64c cysteine protease inhibitors). However, the relative importance of cathepsin B inhibition can be resolved by evaluating E64d in cathepsin B gene-deficient animals. Those studies show that E64d treatment produced, with one exception, similar improvement in neuromotor deficits after trauma as did vehicle controlled treatment of cathepsin B knockout animals (48). Also, the E64d-treated cathepsin B knockout animals tended to have smaller brain lesion volumes and higher neuronal cell densities than vehicle carrier-treated knockout animals but the differences were not statistically significant. As such, those data argue that E64d acts primarily, but perhaps not exclusively, through inhibiting cathepsin B in this TBI model.

The E64d-treated cathepsin B knockout animals performed significantly (20%) better in the neuromotor assay than carrier-treated cathepsin B knockout animals on day 1 post-TBI (48). These results suggest that E64d produced an additional benefit in behavior from inhibiting other proteases in addition to cathepsin B. While it is impossible to say from the data what those other protease(s) are, it is likely that E64c inhibition of calpain contributed to the additional benefit. That is because calpain activity spikes in the first day after TBI (276). Calpain 1 gene-deficient animals have reduced brain lesion volume and neuronal cell death following TBI (277), and E64d has been shown to inhibit calpain activity and provide neuroprotection after trauma (249). Thus, while E64c inhibition of cathepsin B is primarily responsible for the improvements in the TBI models, its ability to inhibit other proteases produces even better outcomes compared to inhibition of only cathepsin B.

Primary benefits due to inhibiting cathepsin B and the additional benefits from inhibiting related proteases have also been demonstrated in ischemic non-human primate models. CA-074, which has absolute specificity for cathepsin B (see below, section on CA-074, selective inhibitor of cathepsin B), given iv immediately after ischemia significantly inhibited brain cathepsin B by about 75% in the CA1 region of the hippocampus and caused substantial neuronal protection by saving about 67% of the neurons in that region relative to controls (108). Studies comparing the effects of CA-074 with that of E64c in the non-human primate model showed that even better outcomes resulted from using E64c (109, 252). For example, E64c and CA-074 treatments resulted in an 84% and 67% CA1 neuronal cell survival, respectively (109), and a range of cell protection from 75% (cortical layer 5) to 91.6% (cerebellum) vs. 47.4% (CA1) to 89.9% (caudate nucleus), respectively, relative to controls (252). The authors attributed the increased neuronal cell survival obtained with E64c over that of CA-074 to E64c inhibition of cathepsin L and calpains in addition to cathepsin B, whereas CA-074 only inhibited cathepsin B.

Another advantage of multiple protease inhibition by E64d is the indirect reduction in matrix metallopeptidase-9 (MM-9), which is a protease that contributes to TBI dysfunction (278). Up-regulation of both cathepsin B and calpain causes up-regulation of MMP-9 (279) and since E64c inhibits both cathepsin B and calpain, E64d treatment also indirectly down-regulates MMP-9 as E64d has been shown to do in an ischemic animal model (280).

The cysteine protease inhibitor, morpholinurea-leucine-homophenylalanine-vinylsulfone-phenyl (LHVS), provides significant improvements in behavioral deficits, brain edema, and cell survival following TBI (248). LHVS was thought to specifically inhibit cathepsin S, which is up-regulated following TBI (248), but LHVS has subsequently been shown to inhibit cathepsin B, S, and L (146). Thus, the beneficial results in the TBI model may be the result of LVHS inhibiting these several proteases. That is the case for the improved outcomes seen in an MS model given LHVS in which the compound has been shown to act by inhibiting both cathepsins B and S (146). E64 has been reported to inhibit cathepsin S (260) and thus E64d treatment may also provide beneficial TBI effects through inhibiting cathepsin S in addition to that achieved by cathepsin B inhibition.

Thus, cathepsin B inhibition is a primary means by which E64d provides beneficial outcomes in TBI and ischemic models, but additional benefits are obtained by the compound’s inhibition of other related proteases, especially calpains, cathepsin L, and cathepsin S. The ability of E64d to inhibit multiple related cysteine proteases is thus an advantage and may be required to “move the needle” and affect outcomes in the complex condition of TBI.

#### Biological Effects of Irreversible Cathepsin B Inhibition

E64 and its related compounds as well as many other cathepsin B inhibitors are irreversible inhibitors meaning that they covalently bind to the traget protease and permanently stop that protease’s activity. As a result, cathepsin B activity lost due to these inhibitors can only be restored through production of new mature cathepsin B protein. Normally, cathepsin B turnover (net synthesis and degradation) as measured by the half-life is about 14 h and results in a lysosomal content of about 330 mg/g in rat macrophages (87, 92). E64 treatment of those cells reduces cathepsin B turnover as the cathepsin B half-life is increased 3.6-fold (50 h) and increases lysosomal content threefold higher (990 mg/g) relative to untreated cells (87, 92). The reduced cathepsin B turnover and increased lysosomal content are due to E64 inhibiting the cysteine proteases which degrade cathepsin B (87). Thus, irreversible inhibition of cathepsin B results in its inactivation as well as increased half-life. New synthesis of cathepsin B will be needed to replenish cellular levels of active cathepsin B.

### Discovery of E64d and related compounds

#### E64, E64d, and E64c Cysteine Protease Inhibitors

The discovery of the epoxysuccinyl-based inhibitor E64 was seminal in the development of cathepsin B inhibitors (257). This natural product of *Aspergillus japonicas* selectively inhibits the papain-like cathepsin proteases (except cathepsin C) and calpains 1 and 2. But E64 does not affect cysteine proteases belonging to the CD clan, which includes caspases, or proteases belonging to the aspartyl, serine or metalloprotease classes or thiol enzymes (70, 257–260). For over 30 years, this scaffold has been used to develop new compounds having medicinal properties and that use continues today (281).

The most important derivative of E64 is E64d (aka EST and Loxistatin), which was developed by Taisho Drug Company, Japan, and the Japanese Ministry of Health in the 1980s as an ­experimental therapeutic agent for treating muscular ­dystrophy (262). The clinical trials in pediatric patients were initiated based on studies in which E64d had some, but not a great deal, of effect in a dystrophic hamster model (282). The trials completed through Phase 3, but did not show sufficient efficacy for muscular dystrophy and the compound did not advance (283).

But importantly, extensive E64d pharmaceutical data were published as a result of this effort in many peer-reviewed Japanese scientific articles showing that the compounds could be safely used in man. Some of those data are summarized below (section on pharmaceutical properties of E64d and safety).

E64d is an ethyl ester prodrug, which is hydrolyzed in the gut to its active acid form E64c, which systemically circulates and is the biologically active inhibitor form (284). E64c has the same inhibition profile as E64, but is more potent (285). For example, the potency (IC_50_) of E64c against rat liver cathepsins L, B, H, and porcine kidney calpain 2 is 0.09, 3.36, 1,640, and 3,000 nM, respectively, with the substrates Z-Phe-Arg-MCA, Z-Arg-Arg-MCA, Arg-MCA, and casein, respectively (264), indicating varying potencies of E64c for inhibition of these cysteine proteases. E64c inhibition of calpain 2 is dependent on pH and calcium ion concentration and pH (286). Thus, oral administration of E64d results in systemic circulation of E64c, which can inhibit cathepsins B, L, K, H, O, S, V, W, and X, and calpains 1 and 2, but not other proteases or thiol-containing enzymes.

#### CA-074, Selective Inhibitor of Cathepsin B, and CA-074Me

A specific inhibitor of cathepsin B was developed from the E64 template, known as CA-074, which irreversibly inhibits with high potency (264, 265). CA-074Me was developed from CA-074 to have better membrane permeability (269) but inhibits both cathepsin B and L (267, 268).

#### E64 Activity-Based Probes (ABPs)

E64 has given rise to activity-based probes (ABPs), which bind to the active site of proteases and report on activities via a biotin or quencher-fluorophore tag (287–289). ABPs have been used to affinity purify cathepsin B activity from regulated secretory vesicles of chromaffin cells to discover its β-secretase activity in those vesicles (148). ABPs can image proteases *in vivo* (254, 290, 291) and would provide powerful visual data on cathepsin B distribution in TBI. ABPs can evaluate a drug’s engagement with cathepsin B (290). ABPs could be adapted to evaluate inhibitor target engagement with brain cathepsin B in animal models and in the clinic.

### Pharmaceutical properties of E64d

#### Oral and Peripheral E64d Administration is Efficacious in Animal Models

An important result from a TBI translational point of view is that oral administration of E64d (one 10 mg/kg dose) has been shown to be efficacious in a TBI animal model when given up to 8 h after trauma (48). That is a key milestone because 8 h represents a time frame in the TBI animal model for drug administration that could be applied post injury in the clinic. In most clinical TBI therapeutic applications, the earliest time when therapy can begin is about 6 h after injury and, thus, it is critical that a compound is effective at times after the TBI injury event in the animal model. Moreover, the data discussed above show that cathepsin B remains elevated for days after injury suggesting that an even longer effective treatment window is likely.

Furthermore, administration of E64d by oral gavage to guinea pigs (1–10 mg/kg/day, 7 days) resulted in dose–response reductions in brain cathepsin B activity and Aβ (256). And feeding E64d (10 mg/kg/day, 1 to 2 months) in chow to transgenic mice also reduced brain Aβ and pGluAβ (141, 256). Moreover, icv E64c (4 mg/kg) administration reduced brain cathepsin B activity in ischemic monkey models (109, 252) and ip E64d administration inhibited brain cathepsin B activity in ischemic rat (5 mg/kg) (106) and epileptic rat (4 μg dose) models (122). Thus, oral and other peripheral routes of E64d administration at doses comparable to that used in man (5 mg/kg/day) have been shown to inhibit cathepsin B and affect brain biomarkers in animal models.

#### E64d/E64c Biodistribution

Radioactive E64d biodistribution studies show that about 60% of an oral E64d dose is taken up in rats and hamsters and most of that occurs in the small intestine. Blood levels quickly rise and the maximum blood concentration varies among animal species with rabbits having the highest, and hamsters, dogs, and rats having 75%, 33%, and 25% that of rabbits, respectively. The excretion also varies among species, with most occuring via the urine in rabbits and hamsters whereas the feces was the primary route in dogs and rats. Biliary excretion was greater than 50% and 13% of the dose in rats and hamsters, respectively. Respiration only accounted for about 7% of the dose (292).

The tissue distribution was similar for rats, rabbits, and hamsters, but the highest drug concentration was in the gastrointestinal track followed by the kidney and liver which had about 25- and 12-fold higher concentrations than the plasma concentration, respectively. But the brain concentration was one-tenth that of the plasma concentration. However, the liver drug concentration in rats was about twice that of hamsters (293).

At a cellular level, subcutaneous radioactive E64c injection of rats resulted in a muscle cytoplasm to lysosome-specific activity ratio of about 5:1 (294). That distribution is advantageous for TBI treatment because the drug is concentrated in the cytoplasm where it is needed in the injury condition and not in the lysosomes where it could interfere with normal proteolysis.

#### E64d Pharmacokinetics and Metabolites

Pharmacokinetic properties of E64d have been determined in animals and man (284, 295, 296, 297). In man, an oral E64d dose is completely hydrolyzed in the gut to the acid form of E64c which systemically circulates and about half of that is metabolized to two hydroxylated E64c metabolites (M4a and M4b). E64c and M4 have about the same area under the curve (AUC) values (5.08 μg-hr/mL), serum half-lives of about 1.34 and 2.5 h, respectively, and urinary excretion rates of 28.9% and 18.5%, respectively. Single and multi-dose pharmacokinetics are the same, the drug does not accumulate and is completely eliminated within 24 h of the last dose in the urine at a recovery rate is about 30%. The M4 metabolites have the same inhibitory profile as E64c. Once a day dosing is effective despite the relatively short half-lives because the compounds are potent irreversible inhibitors.

#### E64d Mutagenesis

*In vitro* E64d mutagenic studies using the reverse mutation (Ames bacterial cell test) and chromosome aberration test (Chinese hamster lung fibroblast cells) showed no mutations or chromosome aberrations. *In vivo* mutagenic studies using the mouse bone marrow chromosome aberration test showed that E64d did not have any effects in that assay. The conclusion was that E64d is unlikely to have mutagenic effects (298).

#### E64d Toxicology

Toxicity studies show that E64d has a wide therapeutic window. Extensive E64d toxicology studies have been published. For example, acute oral E64d toxicity studies showed that high lethal doses to be over 10,000 and 5,000 mg/kg for rats and dogs, respectively, which killed 50% of the animals (LD_50_), with the main clinical effects being depression of voluntary movements, closed eye, and loss of righting reflex, from which it was concluded that acute toxicity is not an issue (299). Longer-term toxicity studies showed the kidneys and liver to be the primary target organs for toxicity and the toxic effects increased with increasing dose and generally abated upon discontinuing the drug. Sub-acute, 1 month oral E64d toxicity studies in dogs and rats found no-effect doses of 40 and 80 mg/kg/day, respectively, with slight renal tubular epithelia degeneration but without an increase in renal weight or impairment of function (300, 301). Six-month chronic oral E64d studies in dogs found a no-dose effect level at 5 mg/kg/day, with similar renal results as in the sub-acute studies (302). Six-month chronic oral E64d studies in rats, on the other hand, found a no-effect dose of 2 mg/kg/day for liver toxicity and10 mg/kg/day for renal toxicity and that the rat was unusually sensitive to liver toxicity (303). Subsequent studies confirmed the rat species-specific hepatotoxicity as a single oral E64d dose produced rat liver toxicity at 60 mg/kg, but in a hamster liver toxicity required a 500 mg/kg dose (297, 304). The rat liver toxicity was attributed to the rat metabolizing the drug differently than rabbits, hamsters, and man because the rat mostly excreted the drug as glutathione conjugates via bile whereas in man and other animals the free drug was mostly excreted via urine (284). Rats have much higher liver glutathione levels, which E64c and M4 metabolites bind to, and thus the rat liver dose is proportionally greater than that in other species (304).

#### E64d Teratogenic Effects

Teratogenic effects have also been studied and also show differences among species. In rabbits, the no teratogenic effect for oral E64d doses was found to be 100 and 500 mg/kg/day for fetuses and mothers, respectively, when administered from the 6th through the 18th day of gestation (305). By contrast, the no teratogenic effect oral E64d dose in rats was 20 mg/kg/day for fetuses and mothers when given at about the same time frame (7th through the 17th day of gestation) (306). Thus, the rat is much more susceptible to E64d teratogenic effects than rabbits. Rat studies using oral dosing before pregnancy and during mating and late-term and post-natal showed the lowest no-effect dose for the fetuses and parents to be about 50 and 80 mg/kg/day (307, 308). No effect was seen in the subsequent generation (F2) in these studies.

### E64d safely used in man

As mentioned above, E64d was developed for treating muscular dystrophy. Phase 1 single (oral 3.5 mg/kg maximum dose) and multi-dose (oral, 5 mg/kg/day for 7 days) studies in normal volunteers and showed no effect on observed clinical symptoms, including electrocardiograms, body temperature, blood pressure, grip strength, and physician interviews, or clinical tests, including hematological tests, blood chemistry tests, and urinalysis (295, 296). These E64d doses used in patients are below the range of doses evaluated in animals for toxicology and teratogenic studies.

Open trials on 73 Duchene’s muscular dystrophy patients were conducted for 3 years at four Japanese national sanatoriums and resulted in some muscle strength improvement but the results were inconclusive and final double-blind studies did not confirm the results (283). The fact that these trials were completed shows that no serious adverse event occurred in the pediatric patient population with long-term chronic administration of E64d.

### Issues with E64d TBI therapy

The gross abnormalities seen in offspring of double cathepsin B and L knockout mice clearly show that some cysteine cathepsin activity is required for normal embryogenesis. Possibly, the loss of cysteine cathepsin protease protein degradation in lysosomes and autophagosomes results in nutritional deficiencies, which cause the abnormalities. As discussed above, sufficiently high E64d doses can also cause teratogenic abnormalities when administered to pregnant animals.

This has consequences for the therapeutic use of E64d or its derivative, the most obvious being that very careful consideration will have to be given to its use in pregnant women, women of child-bearing age, and infants. But as most young civilian and military TBI patients are adolescent or young adult males, the teratogenic danger may not be as much of a risk for the majority of that TBI patient population. Moreover, the teratogenic risk is not likely an issue for a large and growing segment of the TBI population, which are elderly who are beyond the reproductive age.

These data also suggest that minimizing inhibition of lysosomal cathepsin B and cathepsin L activities is desirable in adults. To that end, E64c is unique among cysteine protease inhibitors in that it concentrates in the cytosol and not in the lysosome (see above section on E64d/E64c biodistribution), thus preferentially spares inhibition of lysosomal cathepsin B and L activities. The fact that E64d has been safely administered to pediatric patients for many years shows that, if lysosomal protease inhibition is a toxicity issue, it is not significant with an appropriate dose.

The low brain dose is the primary issue with E64d as a TBI therapeutic because a small portion of an oral dose gets into the brain as discussed above (section on biodistribution). Even though a low brain dose can effectively inhibit brain cathepsin B activity, it would be desirable to have a derivative compound, which delivers a higher brain dose than does E64d so that efficacy could be achieved with reduced systemic exposure.

### E64d is a promising tool compound for preclinical TBI testing and TBI clinical compound development

E64d is unique among the small molecule cathepsin B inhibitors because of the extraordinary amount of data on the compound. No other compound has the extensive information known for E64d on its efficacy, toxicology, and safety. These data greatly reduce the risk of using E64d vs. other small molecule compounds for testing in TBI and related models. Therefore, E64d is a promising tool compound for preclinical TBI testing.

The extraordinary amount of data makes the E64d scaffold extremely useful for developing clinical derivatives. Only the E64d compound has data on pharmaceutical properties that provides an expectation roadmap for the behavior of a clinical compound that is derived from it. Other small molecule scaffolds lack that information and thus all the data on clinical candidates derived from them will be without information as to the likelihood of pharmaceutical success. Thus, the E64d data greatly reduce the inherently high risk of drug development.

One might ask why not use a cathepsin B-specific inhibitor rather than the multi-protease inhibitor E64d since cathepsin B knockouts have no pathology? A druggable cathepsin B-specific inhibitor may be useful as a follow-on therapeutic. However, no orally bioavailable, brain effecting, cathepsin B-specific inhibitor is currently available and developing such will be difficult and will take considerable resources and time. For example, a high throughput screen of the National Institutes of Health (NIH) small molecule library for a cathepsin B-specific inhibitor did not find a druggable hit (309). The known cathepsin B-specific inhibitor, CA-074, is not orally druggable and when made more so with the addition of a methyl group, it looses specificity. Thus, an E64d derivative will likely reach clinical testing much sooner than a cathepsin B-specific compound.

TBI is an urgent unmet need that cannot wait. Furthermore, as discussed above, the multi-cysteine protease inhibition by E64c is more efficacious than selective cathepsin B inhibition and thus E64d derivatives offer a better chance of obtaining positive outcomes in clinical trials.

## Conclusion

Cathepsin B gene deletion studies show that cathepsin B is a key player in the pathology of TBI. Cathepsin B is elevated following TBI and its removal or inhibition dramatically improves outcomes following trauma in animal models. Moreover, deleting or inhibiting cathepsin B improves outcomes in injury models related to TBI including epilepsy, aneurysm, ischemia, pain, surgical trauma, spinal cord trauma, infectious disease, and neurodegeneration. E64d is a promising compound for further preclinical testing as a candidate therapeutic agent for TBI because it has been shown to be orally effective when administered after trauma. E64d is the best scaffold upon which a TBI therapeutic can be developed because only E64d has extensive clinical and preclinical data available. It will also be beneficial to consider that inhibition of cathepsin B targets several mechanisms involved in TBI, including calpain and matrix metalloproteinases that contribute to TBI, as explained in this review. In conclusion, basic and translational research on cathepsin B for improving TBI-caused deficits should be accelerated as it is highly likely to generate an effective new TBI therapeutic.

## Conflict of Interest Statement

Gregory Hook is an employee and has equity in ALSP, Inc., which is developing drugs to treat neurological conditions, including TBI. Vivian Hook is the chair of ALSP’s Scientific Advisory Board and J. Steven Jacobsen and Kenneth Grabstein are members of that Board and all hold equity in ALSP. J. Steven Jacobsen is an employee of AstraZeneca, a major pharmaceutical company. Vivian Hook and J. Steven Jacobsen have disclosed their relationship with ALSP to the University of California, San Diego (La Jolla, CA, USA) and AstraZeneca, respectively. Mark Kindy has no conflict of interest to declare.
